# Reducing the inherent auto-inhibitory interaction within the pegRNA enhances prime editing efficiency

**DOI:** 10.1093/nar/gkad456

**Published:** 2023-05-29

**Authors:** Karthikeyan Ponnienselvan, Pengpeng Liu, Thomas Nyalile, Sarah Oikemus, Stacy A Maitland, Nathan D Lawson, Jeremy Luban, Scot A Wolfe

**Affiliations:** Department of Molecular, Cell and Cancer Biology, University of Massachusetts Chan Medical School, Worcester, MA, USA; Department of Molecular, Cell and Cancer Biology, University of Massachusetts Chan Medical School, Worcester, MA, USA; Department of Molecular Medicine, University of Massachusetts Chan Medical School, Worcester, MA, USA; Department of Molecular, Cell and Cancer Biology, University of Massachusetts Chan Medical School, Worcester, MA, USA; Department of Molecular, Cell and Cancer Biology, University of Massachusetts Chan Medical School, Worcester, MA, USA; Department of Molecular, Cell and Cancer Biology, University of Massachusetts Chan Medical School, Worcester, MA, USA; Department of Molecular Medicine, University of Massachusetts Chan Medical School, Worcester, MA, USA; Li Weibo Institute for Rare Diseases Research, University of Massachusetts Chan Medical School, Worcester, MA, USA; Department of Molecular Medicine, University of Massachusetts Chan Medical School, Worcester, MA, USA; Li Weibo Institute for Rare Diseases Research, University of Massachusetts Chan Medical School, Worcester, MA, USA; Department of Molecular, Cell and Cancer Biology, University of Massachusetts Chan Medical School, Worcester, MA, USA; Li Weibo Institute for Rare Diseases Research, University of Massachusetts Chan Medical School, Worcester, MA, USA

## Abstract

Prime editing systems have enabled the incorporation of precise edits within a genome without introducing double strand breaks. Previous studies defined an optimal primer binding site (PBS) length for the pegRNA of ∼13 nucleotides depending on the sequence composition. However, optimal PBS length characterization has been based on prime editing outcomes using plasmid or lentiviral expression systems. In this study, we demonstrate that for prime editor (PE) ribonucleoprotein complexes, the auto-inhibitory interaction between the PBS and the spacer sequence affects pegRNA binding efficiency and target recognition. Destabilizing this auto-inhibitory interaction by reducing the complementarity between the PBS-spacer region enhances prime editing efficiency in multiple prime editing formats. In the case of end-protected pegRNAs, a shorter PBS length with a PBS-target strand melting temperature near 37°C is optimal in mammalian cells. Additionally, a transient cold shock treatment of the cells post PE-pegRNA delivery further increases prime editing outcomes for pegRNAs with optimized PBS lengths. Finally, we show that prime editor ribonucleoprotein complexes programmed with pegRNAs designed using these refined parameters efficiently correct disease-related genetic mutations in patient-derived fibroblasts and efficiently install precise edits in primary human T cells and zebrafish.

## Graphical Abstract

**Graphical Abstract:**
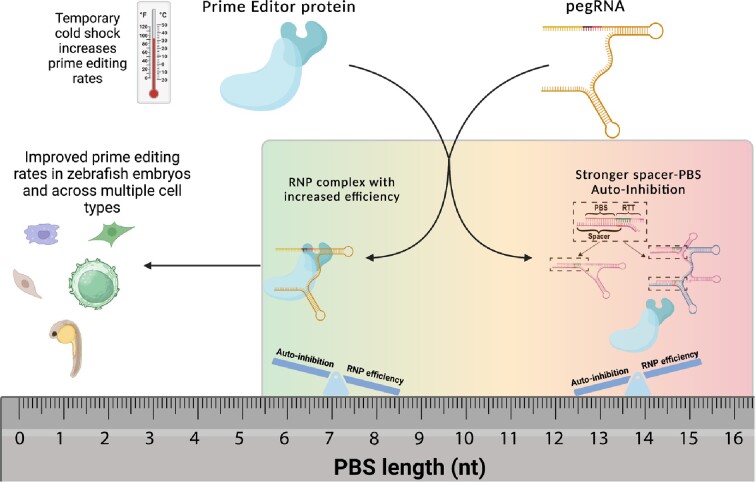

## INTRODUCTION

The correction of genetic mutations *ex vivo* or *in vivo* has broad potential therapeutic application for a range of human genetic diseases. A prime editor (PE) composed of a Cas9 nickase (for SpyCas9-based systems the H840A nickase) and an engineered reverse transcriptase (typically MMLV-RT) can produce precise nucleotide changes, sequence insertions and deletions (1). This innovative technology neither induces double-stranded DNA breaks nor requires a donor DNA template in conjunction with homology-directed repair to introduce precise sequence changes into the genome. Unlike base editing systems, which suffer from the challenge of bystander base conversion in some sequence contexts (2), prime editing systems rewrite local sequences based on a co-delivered RNA template sequence. Consequently, prime editors provide a potentially revolutionary tool for somatic cell genome editing.

Several versions of prime editors were originally described for genome modification (1). The PE2 version utilizes an engineered MMLV-RT along with the Cas9 nickase to rewrite genomic sequences in a targeted manner through a multi-step process: (i) Cas9 target sequence recognition through the spacer sequence encoded at the 5’ end of the pegRNA; (ii) nicking of the non-target DNA strand upon R-loop formation; (iii) annealing of the free 3’ end of the nicked DNA to the primer binding site (PBS) at the 3’ of the pegRNA; (iv) MMLV-RT appending the sequence defined by the reverse transcriptase template (RTT) region of the pegRNA to the free 3’ DNA end and (v) endogenous DNA repair pathways incorporating the extended DNA sequence into the genome, which can be facilitated by sequence homology (homology arm, HA) encoded within the RTT. Multiple approaches can increase precise repair (successful template-dependent prime editing) rates, including: prime editors with improved efficiency (3–6), pegRNA designs with improved stability (7–9), the introduction of a nick (PE3 version) (1) or a second prime editor complex editing the opposite DNA strand (9–13) and inhibition of DNA repair factors (PE4 & PE5 versions) that disfavor the incorporation of prime editor DNA products into the genome (6,14).

Utilization of prime editing systems to enable genome alteration has been primarily focused on DNA (1,4,15), RNA (6,8,16,17) or viral delivery (4,18–20). Prime editor protein - pegRNA complexes (PE RNPs) have been successfully employed in transformed cell lines, zebrafish embryos, primary human T cells and induced pluripotent cells (21,22). However, in general precise editing rates in these studies (21,22) were modest (∼10%) compared to the editing rates that have been achieved by other delivery methodologies (1,8,14). Here, we report that the inherent complementarity between the PBS and spacer sequence within the pegRNA restricts the efficiency of prime editing. This complementarity can extend into the first three nucleotides (nt) of the RTT region if it is identical to the DNA target site. Because RNA–RNA duplexes are typically more stable than RNA–DNA duplexes (23) and due to the intramolecular nature of the association between the spacer and PBS regions of the pegRNA, the formation of a PBS–spacer RNA duplex can prevent the prime editor from forming an R-loop at its target site. Finding the correct balance between PBS length and pegRNA sequence composition is critical to maximize prime editing activity by reducing the inherent ‘auto-inhibition’ within the pegRNA sequence composition. We demonstrate that reducing the complementarity between the PBS and spacer sequences by reducing PBS length can broadly improve prime editing activity in the context of prime editor protein complexed with synthetic, end-protected pegRNAs across multiple target sites and multiple cell types.

## MATERIALS AND METHODS

### General methods and molecular cloning

To generate pegRNA expression plasmids, gblocks or PCR products including spacer sequences, scaffold sequences and 3’ extension sequences (RTT, PBS & pseudoknot) were amplified with indicated primers (Supplementary table) using Phusion master mix (ThermoFisher Scientific, #F-548L). These amplicons were subsequently cloned into the sgRNA, pegRNA or epegRNA U6 expression vectors (Addgene, #122089) by the Gibson assembly method (NEB, #E2611L). To generate sgRNA expression plasmids, annealed oligos were cloned into BfuAI-digested vectors. To generate pegRNA and epegRNA expression plasmids, BfuAI and EcoRI digested vectors were used. Sequences of all pegRNA, epegRNAs and sgRNAs are listed in the Supplementary table. All plasmids used for transfection experiments were purified using PureLink™ HiPure Plasmid Midiprep Kit including endotoxin removal step (ThermoFisher scientific,#K210005). pCMV-PEmax was a gift from David Liu (Addgene plasmid #174820). To generate PEmax protein expression vector (pET-21a-PEmax-6His), Primers were used to amplify the SpCas9-H840A and M-MLV ORFs from PEmax backbone, and then cloned into the bacterial expression plasmid pET-21a vector by Gibson assembly.

### Small RNA sequencing

The immunoprecipitation protocol (IP) was adapted from the ChIP protocol described by the Castillo laboratory (24). HEK293T cells (10^7^ cells) were plated in 10cm culture dishes and transfected with the prime editor components (10 μg of PEmax or Cas9 vector harboring an HA-tag and 5 μg of pegRNA or epegRNA) using Lipofectamine 3000 (Invitrogen, #L3000001), as per manufacturer's instructions. After 48 h the cells were harvested and for the IP of effector-bound RNAs, cross-linked in 1% formaldehyde for 20 min at room temperature. The cells were then lysed using Pierce™ IP Lysis Buffer (Thermofisher scientific, #87788). Immunoprecipitation of the Cas9 or PE RNP was carried out using anti-HA tag antibody—ChIP Grade (Abcam, #ab9110) overnight at 4°C. Antibody bound RNP complexes were isolated using Dyna magnetic beads (Life Technologies, #10004D). The immunoprecipitated RNP complex was then reverse cross-linked overnight at 65°C. DNaseI (NEB, #M0303S) and proteinase K (Thermofisher Scientific, #25530049) treatment was carried out at 37°C to remove the protein and DNA. The RNA (pegRNA or epegRNA) was then purified using the Monarch® RNA Cleanup Kit (NEB, #T2050L). The small RNA library was built by a protocol adapted from the Illumina TruSeq small RNA library protocol described by the Zamore laboratory (25). Detailed protocol for immunoprecipitation and small RNA library preparation can be found in the Supplementary protocol. The small RNA library was then analyzed by deep sequencing.

### In vitro transcription of PEmax mRNA used in HEK293T, fibroblast cells and T cell experiments

PEmax coding region was cloned into an mRNA vector encoding an T7 promoter followed by a 5’ untranslated region (UTR), Kozak sequence, multiple cloning sites (MCS), and a 3’ UTR with a 125-nt poly(A) tail (26). To produce mRNA, the vector was linearized by PmeI (NEB, R0560S)digestion that cleaves after the polyA tail. PEmax mRNA was transcribed from 500 ng purified linearized template using the HiScribe T7 High-Yield RNA Synthesis Kit (NEB, E2040S) with co-transcriptional capping by CleanCap AG (TriLink Biotechnologies, N-7413-5) and full replacement of UTP with N1-Methylpseudouridine-5’-triphosphate (TriLink Biotechnologies, N-1081-5). After 1 h of *in vitro* transcription, the DNA template was digested by 1 μl DNase I (ThermoFisher Scientific, EN0521) for 15 min. Transcribed mRNAs were purified by RNA Clean & Concentrator-25 kit (Zymo Research, R1018) and then the purified mRNA was dissolved in nuclease-free water. The resulting PEmax mRNA was quantified with a NanoDrop One UV-Vis spectrophotometer (ThermoFisher Scientific) and was stored at –80°C.

### PEmax protein purification

PEmax protein purification protocol was adapted from a previously described protocol for 3x-NLS-SpCas9 (27). pET-21a-PEmax-His_6_ (Supplementary Figure S12) was introduced into *Escherichia coli* Rosetta2(DE3)pLysS cells (EMD Millipore, #71403) for protein overexpression. Cells were grown at 37°C to an OD_600_ of ∼0.6, then pre-chilled in an ice bath for 10 min and shifted to 18°C. At an OD_600_ of ∼0.8 the cells were induced for 16 h with IPTG (0.7 mM final concentration). Following induction, cells were pelleted by centrifugation and then resuspended with Nickel-NTA buffer (20 mM TRIS + 1 M NaCl + 20 mM imidazole + 1 mM TCEP, pH 7.5) supplemented with HALT Protease Inhibitor Cocktail, EDTA-Free (100×) (ThermoFisher scientific, #78439) and lysed with LM-20 Microfluidizer (Microfluidics) following the manufacturer's instructions. The protein pellet was then purified with Ni-NTA resin in batch mode and eluted with elution buffer (20 mM TRIS, 500 mM NaCl, 250 mM Imidazole, 10% w/v glycerol, pH 7.5). The PEmax protein was dialyzed overnight at 4°C in 20 mM HEPES, 500 mM NaCl, 1 mM EDTA, 10% w/v (8% v/v) glycerol, pH 7.5. Subsequently, The PEmax protein was step dialyzed from 500 mM NaCl (overnight, 12–18 h) to 200 mM NaCl (2 h; Final dialysis buffer: 20 mM HEPES, 200 mM NaCl, 1 mM EDTA, 10% w/v glycerol, pH 7.5). Next, the PEmax protein was purified by anion and cation exchange chromatography (Cytiva, #17515601 & #17115201) with the columns stacked in series. The anion exchange column was stacked first to remove nucleic acid contaminants. After loading, this column is removed and the PEmax protein is eluted from the cation exchange column by a salt gradient (Buffer A = 20 mM HEPES pH 7.5 + 1 mM TCEP, Buffer B = 20 mM HEPES pH 7.5 + 1 M NaCl + 1 mM TCEP, Flow rate = 5 ml/min, CV = column volume = 5 ml). The primary prime editor protein peak was dialyzed into 20 mM HEPES pH 7.5, 300 mM NaCl and then was concentrated to ∼30uM-70uM using Amicon® Ultra-15 Centrifugal Filter Unit 100k MWCO (Millipore, UFC910024). We observed some protein aggregation during the concentration procedure indicating challenges with protein solubility and therefore for most of our preps we stop the concentration around 30 uM to preserve protein yield.

### *In vitro* cleavage assay conditions

For the Cas9 nuclease cleavage assay with pegRNA, 10 pmol of pegRNA or sgRNA was added to 5 μl of nuclease free water and then 5 pmol of Cas9 in its storage buffer (20 mM HEPES and 150 mM NaCl, pH 7.4) was added to this solution and incubated at room temperature for 20 min for the RNP complex formation. For reactions with competing oligonucleotide, the pegRNA and the competing oligo (50 pmol) were heated together to 95°C and allowed to cool at room temperature for 5 min before complexing with Cas9 nuclease as described above. Following RNP complex formation, 2 μl of CutSmart® buffer (NEB #B6004) and 500 ng of PCR product containing the target sequence was added to the Cas9 RNP. (The PCR products were labelled with Cy5 by performing PCR with a 3’ primer that is Cy5 labelled for experiments in Supplementary Figure S2). Finally, nuclease free water was added to the reaction to bring the total reaction volume to 20 μl. The cleavage reaction was then incubated at 37°C for 20 min followed by proteinase K treatment for 10 min to stop the cleavage reaction and to digest away the Cas9 that is bound to the DNA ends. The reaction was then run on a 2% agarose gel to observe the cleaved products. To examine the relative binding affinity of Cas9 for a pegRNA or sgRNA, we set up an *in vitro* competition-based cleavage assay. Here we first load 5 pmol of Cas9 nuclease with either 10 pmol of mCherry pegRNA or 10 pmol mCherry sgRNA. After allowing the RNP complex to equilibrate at room temperature for 20 min, we add 10 pmol of the competing AAVS1 sgRNA and carry out the *in vitro* digestion of the appropriate PCR product under the same buffer conditions and temperature as described above.

### Culture conditions for immortalized cell lines and patient derived fibroblasts

HEK293T cells and U2OS cells were purchased from ATCC. RPE-1 cells were a gift from the Sharon Cantor lab. A HEK293T based cell line that contains the MECP2 editing locus with some common Rett syndrome mutations was constructed as described in our recent work (46). Patient derived fibroblasts containing the T158M mutation were a gift from the Rett Syndrome Research Trust. All cells were maintained in Dulbecco's Modified Eagle's Medium supplemented with 10% FBS at 37°C and 5% CO_2_ unless otherwise noted.

### Transfection of HEK293T and U2OS cells

To define unsaturated prime editing conditions for comparison of the activity of various pegRNAs, a series of prime editing reactions were tested where the amount of PEmax plasmid (100, 200, 400, 600, 800, 1000 ng) and pegRNA plasmid (50, 100, 200, 300, 400, 500 ng) were delivered by transfection to HEK293T cells keeping the ratio of PEmax:pegRNA at 2:1 (Supplementary Figure S3a). A ratio of 200 ng PEmax plasmid to 100 ng pegRNA was chosen for editing activity comparisons. For transfection-based editing experiments, HEK293T and U2OS cells were plated 40000 cells per well in a 48-well plate. 24 h later, the cells were co-transfected with 200 ng of prime editor plasmid, 100 ng of pegRNA plasmid. Lipofectamine 3000 (Invitrogen, #L3000001) was used for the transfection according to the manufacturer's instructions. To determine editing rates at endogenous genomic loci, cells were cultured 3 days following transfection, after which the media was removed, the cells were harvested, and genomic DNA was isolated using QIAamp DNA mini kit (QIAGEN, #56604) according to the manufacturer's instructions. The editing rates were then determined by targeted amplicon deep sequencing or by a flow cytometry in the case of the mCherry or TLR-MCV1 reporter lines (Supplementary Figure S13).

### Electroporation of HEK293T, U2OS, RPE-1 and fibroblast cells

PEmax mRNA - sgRNA mixtures or RNPs were delivered by electroporation using the NEON Nucleofection System 10 μl kit (Invitrogen, MPK1096). For PEmax mRNA editing experiments, 100k cells were pelleted at 300 g for 5 min and resuspended in NEON Buffer R. The cell solution was combined with a mixture of 1 μg PEmax mRNA, 100 pmol synthetic pegRNA (IDT) (Supplementary table) in R buffer from the NEON nucleofection kit (Invitrogen, MPK1096). In case of a PE3 editing approach, 50 pmol of synthetic nicking sgRNA (IDT) was added to the mixture of 1 μg PEmax mRNA and 100 pmol of synthetic pegRNA (IDT). The NEON Nucleofection System (Invitrogen) was used for electroporation with 10 μl tips (HEK293T: 1150 V, 20 ms, 2 pulses; U2OS: 1200 V, 20 ms, 2 pulses; RPE-1: 1350 V, 20 ms, 2 pulses and fibroblasts: 1200 V, 30 ms, 2 pluses). For RNP based editing experiments, 50 pmol of PEmax protein was incubated with 200 pmol of pegRNA and 15 pmol of nicking sgRNA (150 pmol of PE protein with 600 pmol of pegRNA and 45 pmol of nicking sgRNA were used in case of fibroblasts) in R buffer to a total volume of 10 μl for 15 min at room temperature. Then 100k cells were electroporated with 10 μl of PEmax RNP complex using the same electroporation conditions described above for mRNA nucleofection. gDNA was isolated 3 days after electroporation from each group and stored at –80°C for Illumina library preparation.

### Prime editing experiments in human primary CD4^+^ T cells

To generate primary CD4^+^ T cells, peripheral blood mononuclear cells (PBMCs) were isolated from human donor leukopaks (source) by gradient centrifugation on lymphoprep (Stemcell Technologies, cat#07861). Thereafter, PBMCs were depleted of CD14 mononuclear cells using anti-CD14 microbead antibodies (Miltenyi Biotec, cat#130–050-201) and the flowthrough was enriched for CD4^+^ T cells by positive selection using anti-CD4 microbead antibodies (Miltenyi Biotec, cat#130-045-101). CD4^+^ T cell enrichment was confirmed by determining the percentage of CD3^+^/CD4^+^ cells via flow cytometry. Isolated CD4^+^ T cells were cultured in complete RPMI-IL2 media (RPMI-1640 media (Thermofisher Scientific, cat #11875093) supplemented with 10% heat-inactivated Cosmic Calf Serum (GE lifesciences, cat #SH30087.03), 25 mM HEPES pH 7.2 (Corning, cat #25–060-CI), 20 mM GlutaMAX (Gibco, cat#3505-061), 1 mM Sodium pyruvate (Corning, cat#25-000-CI), 1X MEM non-essential amino acids (Corning, cat#25–025-CI), 1% penicillin-streptomycin(Gibco, cat#15140-122), and 1:2000 human interleukin-2 (made in-house from IL-2 expressing cell line). Three days prior to electroporation, primary CD4^+^ T cells were activated with anti-CD3/CD28 antibodies (Stemcell Technologies, cat#10971). 150 pmol of PE protein with 600 pmol of pegRNA (IDT) and 45 pmol of nicking sgRNA (IDT) were used for RNP complex formation. 1e6 activated primary CD4^+^ T cells were electroporated with prime editing RNPs using the P3 primary cell nucleofector kit (Lonza Biosciences, cat #V4XP-3032) and program EH-115 on an Amaxa 4D-Nucleofector. The CD4^+^ T cells were allowed to recover for 72 h at 37°C with or without cold shock before genomic DNA is extracted using the Qiagen QiAamp DNA Blood Mini Kit (Qiagen, cat #51104).

### Cold shock treatment for cells post-electroporation

Post nucleofection of the PE mRNA or PE RNP, the cells were moved to an incubator set at 30°C and 5% CO_2_ for 12–16 h. After which, the cells were moved back to 37°C and 5% CO_2_. 72 h post nucleofection, genomic DNA was harvested from the cells using the QIAamp DNA mini kit (QIAGEN, #56604).

### Zebrafish prime editing experiments

Zebrafish were maintained and bred according to standard protocols set by University of Massachusetts Chan Medical School Institutional Animal Care and Use Committee. Zebrafish embryos obtained from EK (WT) wild-type in-crosses were used for one cell-stage microinjections of PE RNPs. Prior to injections the *tek* target sequence was verified by Sanger sequencing. For PE2, 12 μM pegRNA (synthesized by IDT) and 6 μM PE protein were combined in nuclease-free water. For PE3 a nicking sgRNA (synthesized by IDT) was added to the PE2 complex at a 1 to 10 nicking sgRNA to pegRNA molar ratio. Complexes were incubated at room temperature for 5 min and then 2 nl was injected into single-cell embryos. Injected embryos were incubated at 28.5°C overnight. Twenty-four h post injection embryos were assessed for toxicity and genomic DNA was extracted from 20 normally developing embryos using the Qiagen DNeasy Blood and Tissue kit (Qiagen, #69506). Injections were performed in three independent replicates.

### Targeted amplicon deep sequencing to assess editing rates

Genomic DNA was isolated for prime editing analysis from treated cells or zebrafish embryos. Genomic loci spanning each target site were PCR amplified with locus-specific primers carrying tails complementary to the Truseq adapters. 200 ng of genomic DNA was used for the first PCR using Phusion master mix (ThermoFisher Scientific, # F-548L) with locus specific primers that contain i5 or i7 complementary tails. PCR products from the first PCR were used for the second PCR with i5 primers and i7 primers to complete the adaptors and include the i5 and i7 indices. All primers used for the amplicon sequence are listed in Supplementary table. PCR products were purified with Ampure beads (0.9× reaction volume) and eluted with 25 ul of TE buffer and were quantified by Qubit. Equal molar ratios of each amplicon were pooled and sequenced using an Illumina Miniseq. Amplicon sequencing data was analyzed with CRISPResso (https://crispresso.pinellolab.partners.org/) (28). Briefly, demultiplexing and base calling were both performed using bcl2fastq Conversion Software v2.18 (Illumina, Inc.), allowing 0 barcode mismatches with a minimum trimmed read length of 75. Alignment of sequencing reads to each amplicon sequence was performed using CRISPResso2 in standard mode using the parameters ‘‘-q 30’’. For each amplicon, the CRISPResso2 quantification window was positioned to include the entire sequence between pegRNA- and nicking sgRNA-directed Cas9 cut sites, as well as an additional 10 bp beyond both cut sites. For quantification of PE activity at the target site, editing efficiency was calculated as the percentage of reads with the desired edit without indels (‘‘-discard_indel_reads TRUE.’’ mode) out of the total number of reads ((number of desired edit-containing reads)/(number of reference-aligned reads)). For all experiments, other editing outcomes (including indels and imprecise prime editing) was calculated as the number of discarded reads divided by the total number of reads ((number of indel-containing reads)/(number of reference-aligned reads)).The intended editing rate is the number of reads containing precise prime editing out of the total number of reads.

### Statistical analyses

Statistical analyses for plotted data were performed using GraphPad Prism 8.4. In all studies, data represent biological replicates (*n*) and are depicted as mean ± s.d. as indicated in the figure legends. Comparison of mean values was conducted with unpaired (except for Supplementary Figure S11), two-tailed Student's *t*-test; one-way ANOVA; or two-way ANOVA with Tukey's multiple comparisons test, as indicated in the figure legends. In all analyses, *P* values <0.05 were considered statistically significant.

### Data availability/sequence data resources

Illumina Sequencing data have been submitted to the Sequence Read Archive. These datasets are available under BioProject Accession number PRJNA907921 (https://www.ncbi.nlm.nih.gov/bioproject/?term=PRJNA907921) (SRA number: SRR23012416–SRR23012421). The authors declare that all other data supporting the findings of this study are available within the paper and its Supplementary Information files or upon reasonable request. Backbone plasmids used for pegRNA and sgRNA cloning are available from Addgene (#122089). The PEmax protein expression vector will be deposited with Addgene.

### Web sites/data base referencing/programs

CRISPResso (https://github.com/pinellolab/CRISPResso2)

BWA (v0.7.17) (https://github.com/lh3/bwa)

Samtools (v1.16.1) (https://github.com/samtools/samtools)

umi_tools (v1.1.2) (https://umi-tools.readthedocs.io/en/latest/)

Aravind J, Krishna GK (2022). rmelting: R Interface to MELTING 5. R package version 1.14.0 (https://aravind-j.github.io/rmelting/)

GraphPad Prism 8.4

## RESULTS

### The PBS and spacer region interaction within the pegRNA limits prime editing activity

**Figure 1.**
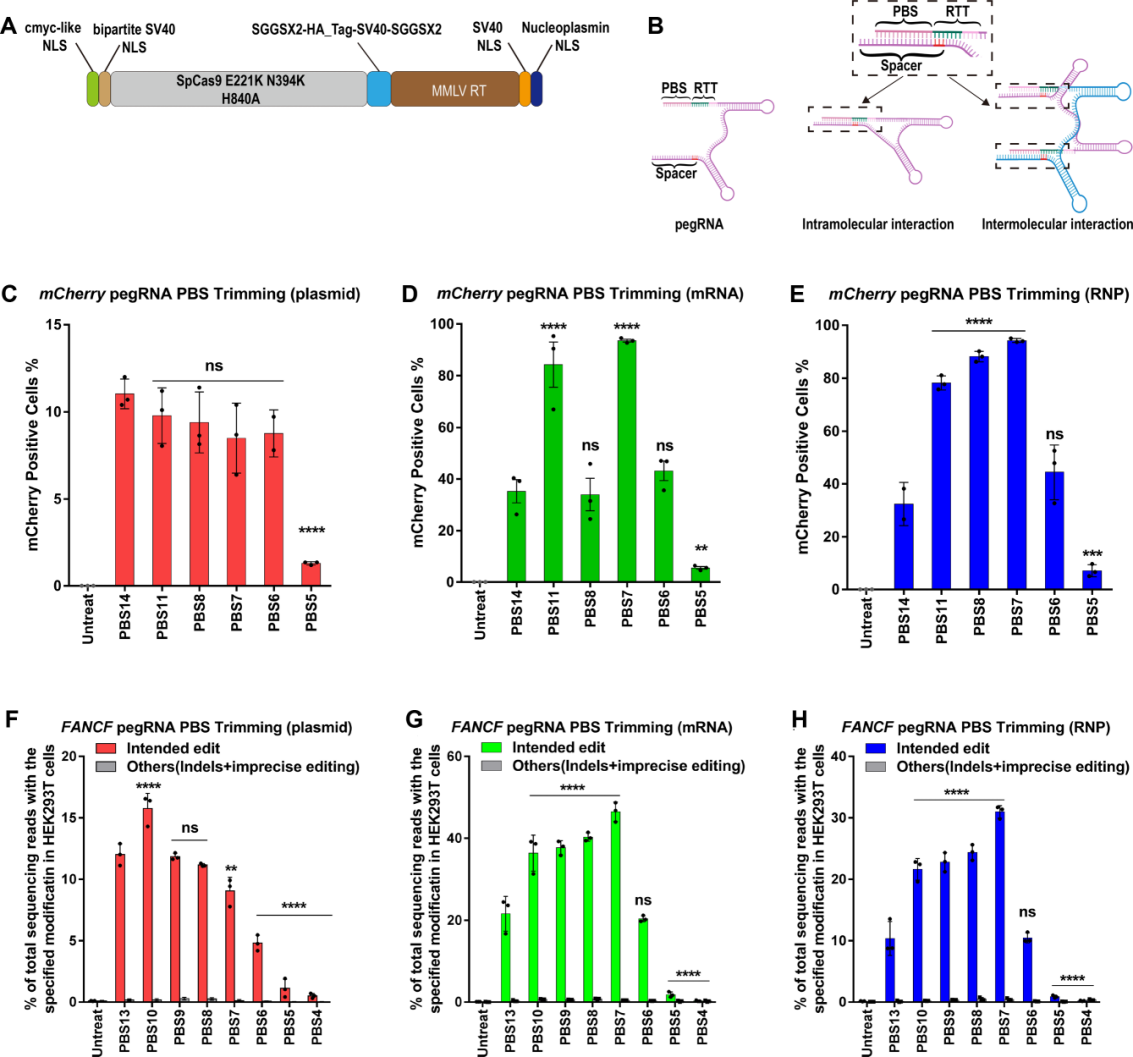
PE2 editing efficiency as a function of pegRNA PBS length at three genomic sites in HEK293T cells using three different delivery platforms. (**A**) Architecture of the PEmax protein expression construct. (**B**) The PBS and spacer sequence within the pegRNA are complementary to each other and can potentially form intramolecular and intermolecular interactions through Watson–Crick base pairing. The complementarity can extend into the first 3 nt of the RTT region if it is identical to the DNA target site (this region is highlighted in the spacer sequence in red). (**C–E**) Conversion of a stop codon (TAG) to glutamine (CAG) by prime editing to restore function to a mCherry reporter in HEK293T cells (4). Frequencies of mCherry positive cells were quantified by flow cytometry 72 h following treatment. (**C**) 200 ng PEmax plasmid and 100 ng pegRNA plasmid were used for transient transfection; (**D**) 1 μg PEmax mRNA and 100 pmol pegRNA were used for mRNA nucleofection; and (**E**) 50 pmol PEmax protein and 200 pmol pegRNA were used for RNP nucleofection. One-way ANOVA was used to compare all the groups for each graph, PBS14 was used as a control column for multiple comparisons. ns indicates *P* > 0.05, ** indicates *P* ≤ 0.01, *** indicates *P* ≤ 0.001 and **** indicates *P* ≤ 0.0001 (also see Supplementary table). (F–G) PE-specified intended substitution (G•C to T•A transversion) at the +5 position of FA Complementation Group F (FANCF) site or other editing outcomes (indels and imprecise prime editing is combined). (**F**) 200 ng PEmax plasmid and 100 ng pegRNA plasmid were used for transient transfection; (**G**) 1 μg PEmax mRNA and 100 pmol pegRNA were used for mRNA nucleofection; and (**H**) 50 pmol PEmax protein and 200 pmol pegRNA (from IDT) were used for RNP electroporation. Cells were harvested 72 h following treatment. One-way ANOVA was used to compare the intended edit across all the groups for each graph, PBS13 was used as a control column for multiple comparisons. ns indicates *P* > 0.05, ** indicates *P* ≤ 0.01, and **** indicates *P* ≤ 0.0001 (also see Supplementary table).

The bacterial expression and purification of prime editor (PE) protein has been described by the Joung and Yeh laboratories (21). We made modifications to the nuclear localization signal (NLS) sequences within the standard PE protein to improve its nuclear localization potential and included two additional point mutations from the PEmax architecture to improve the nickase activity (4,6,27). We then expressed and purified the PE protein from bacteria (Figure 1A, Supplementary Figure S1a). We complexed the purified PE protein with synthetic, end-protected pegRNAs (PE RNPs) that were designed based on sequence composition parameters recommended by prior studies (1,29). However, initial tests of PE RNPs delivered by electroporation to HEK293T cells yielded modest precise editing rates when employing pegRNAs with PBS lengths ∼13 nt (Supplementary Figure S1b–e). Previous studies using plasmid or lentiviral expression systems defined an optimal PBS length for the pegRNA of ∼13 nt in mammalian cells when the A•T and G•C distribution is relatively uniform (1,29). pegRNAs under those assay conditions were expressed endogenously via a U6 promoter and are subject to 3’ degradation (8). The PBS sequence is present at the 3’ end of the pegRNA, and so could be susceptible to truncation. We hypothesized that, in the case of chemically synthesized, end-protected pegRNAs, the PBS length requirements for optimal prime editing activity would be different from plasmid expressed pegRNAs. In particular, the optimal PBS length would reduce the complementarity between the spacer-PBS region to increase the rate of target recognition, nicking and RT priming.

To test the level of auto-inhibition that is inherent in pegRNA structure (7) (Figure 1b, Supplementary Figure S2a), we performed an *in vitro* DNA cleavage assay with SpCas9 nuclease complexed with synthetic pegRNAs. SpCas9 programmed with a pegRNA containing a standard 13 or 14 nt PBS was inactive for DNA cleavage (Supplementary Figure S2b, c). Inhibition was due to the PBS sequence, as introduction of an oligonucleotide complementary to the PBS-RTT region of the pegRNA restored DNA cleavage activity (Supplementary Figure S2a–c). Interestingly, including a competing oligonucleotide that is complementary only to the PBS region was not sufficient to overcome the auto-inhibition interaction at the concentration tested, which may be due in part to additional homology between the last three nucleotides of the RTT and spacer sequence and the intramolecular interaction of the spacer-PBS region within the pegRNA. Thus, *in vitro*, the cleavage activity of Cas9 can be restricted by the pegRNA sequence composition within the PBS region.

### Synthetic pegRNAs with shorter PBS lengths increase prime editing efficiency of PE RNPs at endogenous loci

To examine the impact of the auto-inhibition interaction between the spacer and PBS sequence on PE activity, we tested the editing efficiency of a series of pegRNAs with different PBS lengths. We performed initial tests of these pegRNA designs in HEK293T cells on an mCherry reporter that contains a premature TAG stop codon that prevents translation of a functional protein (4) (Supplementary Figure S1b). We evaluated the prime editing efficiencies in the PE2 format for pegRNAs with different PBS lengths using three different delivery platforms (transfection of expression plasmids encoding the prime editor and pegRNA, or electroporation of PE mRNA or RNP with synthetic pegRNA). Consistent with prior studies (1) for plasmid-encoded prime editor components, the 14 nt PBS had the highest editing efficiency (Figure 1C). However, for PE mRNA or RNP delivered with synthetic pegRNAs, shorter PBS lengths provided higher activity, where the 7 nt PBS afforded the highest prime editing efficiency for both mRNA- and RNP-based systems (Figure 1D and E). Consistent with the increased prime editing rates observed when employing pegRNAs with a shorter PBS length, Cas9 nuclease activity in the *in vitro* DNA cleavage assay was also increased with these pegRNAs suggesting that auto-inhibition is reduced by the shorter PBS-spacer complementarity (Supplementary Figure S2d).

Motivated by our observations in the mCherry reporter cell line, we designed a series of pegRNAs with different PBS lengths for the previously described nucleotide substitution (+5 G to T) at the FANCF locus (1,21) (Supplementary Figure S1c). We observed the highest prime editing efficiency in the PE2 format when using a plasmid expression system under unsaturated conditions for the pegRNA with a 10 nt PBS (Figure 1F, Supplementary Figure S3a), whereas the highest prime editing efficiency when delivering PE mRNA or RNP with a synthetic pegRNA occurred with a 7 nt PBS (Figure 1G, H, Supplementary Figure S3d). Consistent with the prime editing activity outcome, the *in vitro* DNA cleavage assay using Cas9 nuclease programmed with the 7nt PBS pegRNA targeting the FANCF site displayed higher activity (Supplementary Figure S2c).

To determine if the observed trend for PBS length applies to other target site sequence compositions for PE RNPs, we evaluated the optimal PBS length for prime editing activity at two A/T-rich endogenous target sites, MECP2 and BCL11A. At MECP2 we used PE2 to correct a common point mutation (T158M) associated with Rett syndrome, an X-linked neurological disorder (30). The pegRNA PBS length series included a longer PBS (17 nt) based on the design parameters described by Anzalone and colleagues for A/T-rich target sites (1). Consistent with our prior evaluation of PE RNPs programmed with synthetic pegRNAs, shorter PBS lengths displayed higher rates of precise repair with a 10 nt PBS achieving maximum efficiency (Supplementary Figure S4a, b). At BCL11A we used PE2 to disrupt the GATA1 binding motif within the *BCL11A* erythroid enhancer that results in the induction of fetal γ-globin in erythroid progenitors and can ameliorate β-globinopathies like sickle cell disease and β-thalassemia (27,31). We designed pegRNAs with different PBS lengths designed to delete 3 bp from the GATA1 binding motif. Again, our results showed that for PE2-type RNPs, a pegRNA with a shorter PBS length (10 nt) creates the 3bp deletion more efficiently than the pegRNA with a longer PBS length (Supplementary Figure S4a, c). Together, these results suggest that pegRNAs with shorter PBS lengths can broadly improve PE efficacy when employing an RNP format programmed with synthetic pegRNAs.

### Shorter PBS lengths are preferred for plasmid expression systems that generate 3’ end protected epegRNAs

Based on our observation that the prime editor mRNA and RNP systems achieve higher rates of editing with shorter PBS lengths than plasmid expression systems (Figure 1c-h), we speculated that this dichotomy arises from the susceptibility of plasmid-expressed pegRNAs to 3’-exonuclease degradation (8). To address the 3’ degradation issue, the Liu laboratory developed an engineered pegRNA (epegRNA) wherein they appended a 3’ pseudoknot structure to stabilize the pegRNA sequence, which increased the efficiency of prime editing (8). They demonstrated by northern blot that although both pegRNAs and epegRNAs produced from plasmid expression systems are truncated in cells to varying degrees to a species of similar length to an sgRNA, epegRNAs are more stable than pegRNAs when exposed to cell lysates containing exonucleases. We hypothesized that the optimal PBS length would be shorter for epegRNAs produced from a plasmid expression system since they are 3’ end-protected similar to chemically synthesized pegRNAs. To explore the impact of PBS length on prime editing efficiency with epegRNAs, we built two epegRNA plasmid expression vectors for the FANCF target site (FANCF + 5G→T), one with a 13 nt PBS and another with a 7 nt PBS. We observed higher precise editing rates for the epegRNA with the 7 nt PBS, which is consistent with the observations of prime editing with the chemically synthesized pegRNA at this site (Supplementary Figure S3e). Similarly, prime editing with an epegRNA containing a 7 nt PBS was superior to its longer PBS counterpart when targeting the stop codon in the mCherry reporter cell line (Supplementary Figure S3f). Thus, two different forms of pegRNA 3’ end-protection (chemical modification and RNA pseudoknot) yield similar changes in the optimal PBS length for prime editing.

### 3' truncated species compete with full length pegRNA for loading onto the prime editor protein

**Figure 2.**
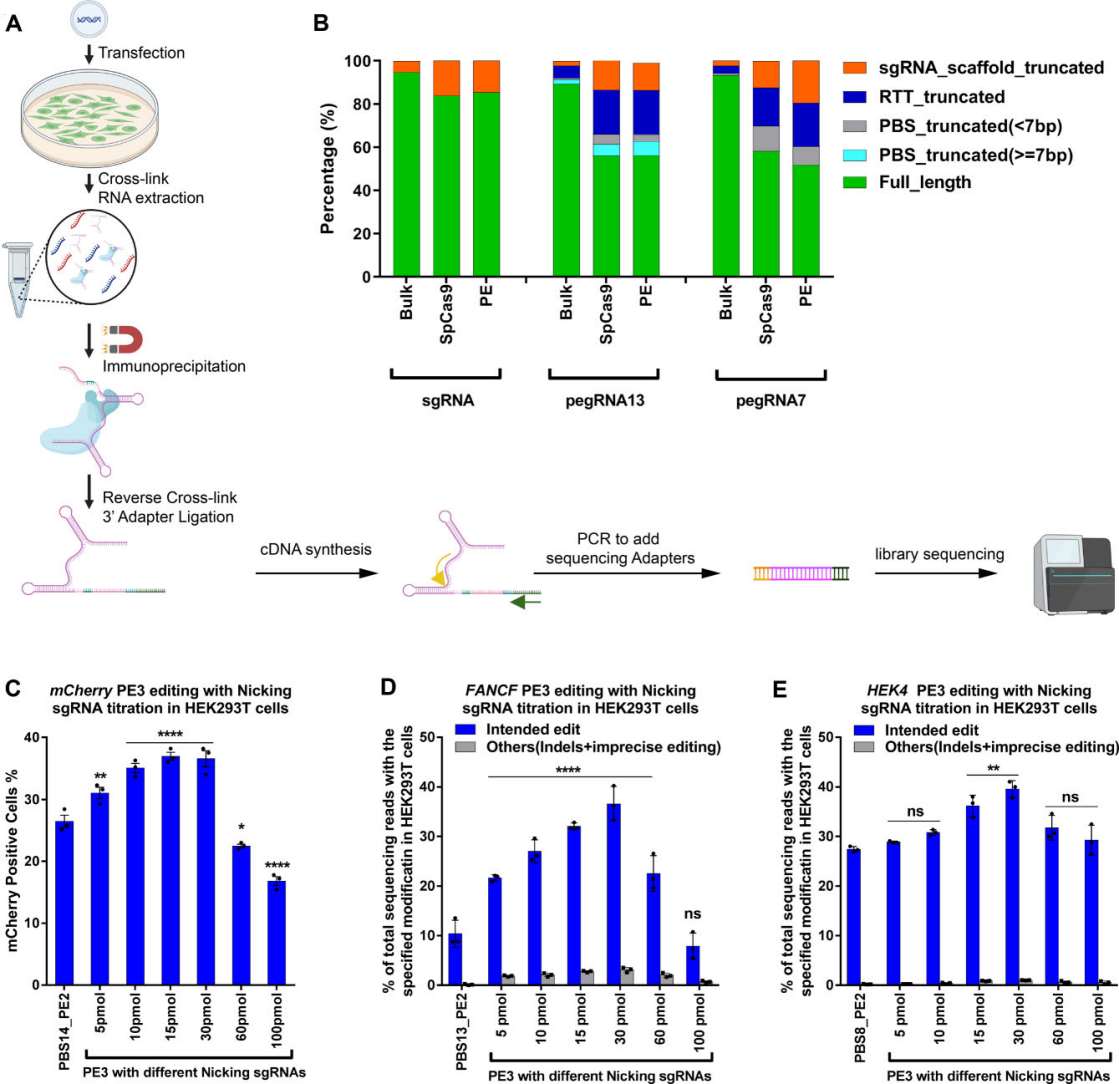
Small RNA-seq analysis of different pegRNA species bound to the Prime editor in HEK293T cells. (**A**) Schematic for the small RNA-seq library preparation. Briefly, HEK293T cells were transfected with plasmids encoding one of two effectors (SpCas9 or PEmax), and one guide RNA (sgRNA, pegRNA or epegRNA). Cells were harvested after 2 days, crosslinked and then lysed for total RNA isolation. To sequence the bound pegRNA or epegRNA population, the SpCas9 or PEmax protein (containing 3xHA-tag) was immunoprecipitated then crosslinking was reversed to purify the bound RNA. This was followed by 3’ DNA adapter ligation (3’ adapter contains 15 bp UMI sequence) to the purified RNA, cDNA synthesis and two rounds of PCR to add sequencing adapters. The final library was deep sequenced and analyzed. A detailed protocol is present in the methods section. (**B**) Bulk or effector-bound RNA species present from each treatment group. ‘Bulk’ indicates sequencing of the sgRNA/pegRNA present in the cell without IP pulldown to examine the sgRNA/pegRNA 3’ sequence lengths irrespective of whether it is bound to SpCas9 or PEmax. The length of the PBS in the pegRNA (7 or 13 nt) is indicated in the name. Small RNAs were categorized into six species based on the length of 3’ truncation: full-length pegRNA, pegRNA with truncated but potentially functional PBS (≥7 nt remaining), pegRNA with truncated likely insufficient PBS (<7 nt), pegRNA with truncated RTT, and pegRNA with truncated sgRNA scaffold. Abundance of each RNA species was calculated based on UMIs incorporated into the 3’ adaptor from the small RNA-seq library (see Supplementary Figure S7 for IGV plots). (**C**) RNP-mediated PE3 editing efficiencies in mCherry reporter cell line with different ratio of pegRNA:nicking sgRNA. The amount of PEmax protein (50 pmol) and pegRNA (200 pmol; IDT) was held constant while increasing the amount of nicking sgRNA (IDT) delivered by electroporation. Frequency of mCherry positive cells was quantified by flow cytometry 72 h following treatment. One-way ANOVA was used to compare all the groups for each graph, PE2 was used as a control column for multiple comparisons. ns stands for *P* > 0.05, * indicates *P* ≤ 0.05,** indicates *P* ≤ 0.01, and **** indicates *P* ≤ 0.0001 (also see Supplementary table). (D, E) RNP-mediated PE3 editing efficiencies at the specified positions for (**D**) FANCF (+5 G to T) and (**E**) HEK4 (+5 G to T) loci in HEK293T cells. The amount of PEmax protein (50 pmol) and pegRNA (200 pmol; IDT) was held constant while increasing the amount of nicking sgRNA (from IDT) delivered by electroporation. Editing efficiency reflects the frequency of sequencing reads from amplicon deep sequencing that contain the intended edit or others (indels and imprecise prime editing) among all sequencing reads. Values and error bars reflect mean ± s.d. of *n* = 3 independent biological replicates. One-way ANOVA was used to compare the intended edit across all the groups for each graph, PE2 was used as a control column for multiple comparisons. ns indicates *P* > 0.05, * indicates *P* ≤ 0.05,** indicates *P* ≤ 0.01, and **** indicates *P* ≤ 0.0001 (also see Supplementary table).

The 3’ truncation of pegRNAs or epegRNAs expressed from plasmid could produce a distribution of species with different lengths. To examine the distribution of 3’ sequence lengths for U6 promoter-expressed pegRNA and epegRNA species and their relative loading distribution on prime editors, we performed small RNA-seq analysis on the total pegRNA and epegRNA population within the cell (Bulk), and of the pegRNA and epegRNA bound to the immunoprecipitated prime editor protein (Figure 2A). To eliminate the possibility that the RNaseH activity of MMLV-RT participates in the truncation of the pegRNA and epegRNA, we also performed pegRNA immunoprecipitation with Cas9 nuclease. Small RNA-seq on the bulk pegRNA and epegRNA species revealed that the majority of products were full-length or nearly full-length (Figure 2B, Supplementary Figure S6, Supplementary Figure S7). However, we observed that the distribution of prime editor (or SpCas9) bound pegRNA and epegRNA species were enriched for truncated products. For both 13 nt PBS and 7 nt PBS pegRNAs, there is an increase in the fraction of truncated products that are bound relative to the bulk populations (Figure 2B). For the 13 nt PBS epegRNA, only ∼30% of the population loaded on the prime editor protein contain the intact PBS, whereas for the 7 nt PBS epegRNA, ∼60–80% of the population loaded on the prime editor protein contain the intact PBS (Supplementary Figure S6, Supplementary Figure S7). In fact, a greater fraction of bound truncated species was observed for the epegRNAs than the pegRNAs (Figure 2B, Supplementary Figure S6, Supplementary Figure S7). This could be in part because an epegRNA has lower binding affinity than a pegRNA to Cas9 (8). Regardless, it is evident that truncated species compete with full-length pegRNA for binding to the prime editor protein or Cas9 nuclease, which would reduce the number of active prime editing complexes and could produce non-productive editors that can bind and nick the target site but cannot initiate prime editing.

### The ratio of nicking sgRNA and pegRNA affects the efficacy of PE3

Since we observed that the truncated species outcompete full length pegRNAs, we questioned whether this would also affect the PE3 approach when an sgRNA is co-delivered with the pegRNA. This could especially affect PE3 RNP complex formation where the amount of PE protein provided is limited and guide RNAs are typically provided in excess. To test if the sgRNA will load on to the protein preferentially over the pegRNA, we designed an *in vitro* competition-based cleavage assay (Supplementary Figure S5a). We loaded Cas9 nuclease with an excess of either mCherry pegRNA or mCherry sgRNA to form their respective RNP complexes. Next, a competing sgRNA targeting the *AAVS1* locus was added to the binding reaction before carrying out the *in vitro* digestion reaction with either the mCherry or AAVS1 target site. Since Cas9 cleavage of DNA *in vitro* is end-product inhibited (32), the amount of Cas9 complex loaded with each guide RNA can be assessed in the presence of excess DNA target. If the Cas9 nuclease has a lower binding affinity for the pegRNA compared to the sgRNA, the AAVS1 sgRNA will probably be preferentially bound to Cas9 even when preloaded with the mCherry pegRNA. When the mCherry sgRNA is pre-equilibrated with Cas9 and then competed with the AAVS1 sgRNA, the resulting complex has only modest cleavage activity on an AAVS1 PCR product. However, when the mCherry pegRNA is pre-equilibrated with Cas9 and then competed with the AAVS1 sgRNA, it cleaves the AAVS1 PCR product to a significantly greater extent (Supplementary Figure S5a,b). These data indicate that the binding affinity of Cas9 protein for the pegRNA is reduced relative to that of an sgRNA targeting the same locus.

Our *in vitro* data demonstrate that an sgRNA is preferentially bound by Cas9 over a pegRNA. Given that both an sgRNA and pegRNA are present in the context of PE3-type prime editing, the ratio of sgRNA to pegRNA may be particularly important to avoid saturation of the available prime editor protein by the sgRNA to the exclusion of the pegRNA. Consequently, we performed experiments to empirically determine the optimal ratio of the pegRNA and nicking sgRNA for maximal activity. Based on our prior analysis for the optimal ratio of pegRNA to PE protein in the absence of nicking sgRNA at two different target sites (FANCF and HEK293T site 4 (HEK4)) (Supplementary Figure S3b, c), we kept the pegRNA to PE protein ratio constant at 4:1 and titrated the amount of nicking sgRNA to determine its optimal stoichiometry for PE3 editing. We tested PE3 editing in the mCherry reporter cell line (Figure 2C) and at two endogenous target sites, FANCF and HEK4 in HEK293T cells (Figure 2D, E). The PE protein was kept constant at 50 pmol and the pegRNA at 200 pmol. The nicking sgRNA concentration was varied from 5 pmol to 100 pmol. We observed that the PE3 system produced precise edits with the highest efficiency when a substoichiometric amount of nicking sgRNA is employed (15–30 pmol). At higher concentrations of the nicking sgRNA, the overall prime editing rate falls, which could be due to the sgRNA displacing the pegRNA across the majority of the delivered PE protein and thereby reducing the number of functional complexes for prime editing.

Recent studies have shown that mismatch repair (MMR) negatively influences prime editing outcomes (6,14). Given that HEK293T cells are partially MMR impaired (33), shifting prime editing to therapeutically relevant cell types that are proficient in MMR may reduce the rate of the desired editing outcome. To confirm that our PBS length analysis and optimal PE3 conditions for PE RNPs translate from HEK293T cells to other cell types where MMR is intact, we tested PE2 editing using the FANCF panel of pegRNAs with different PBS lengths (Supplementary Figure S5c) and PE3 editing at the FANCF and HEK4 loci in U2OS cells (34) (Supplementary Figure S5d, e). We observed that prime editing outcomes in U2OS cells for pegRNAs with different PBS lengths and different nicking sgRNA stoichiometries followed a similar trend as observed in HEK293T cells, albeit with lower precise editing rates.

### Tm of the PBS:spacer DNA determines the optimal PBS length

Consistent with prior models for PBS design (1,29), the optimal PBS length for precise editing was longer for the two A/T-rich target sites tested (MECP2 and BCL11A) than the two G/C-rich target sites (FANCF and mCherry). Using the MELTING 5 program (35), we estimated the melting temperature (Tm) of the optimal PBS sequence with the nicked target DNA for each pegRNA-target site combination. Surprisingly, we found that the estimated Tm of each PBS-target site combination for the optimal PBS length approaches 37°C, which is the growth temperature for mammalian cells (Supplementary Figure S8a-d). To confirm the utility of Tm as a PBS design parameter, we compared prime editing activity using a pegRNA targeting a traffic light reporter system (TLR-MCV1) (36) based on standard design parameters (13 nt PBS) with a pegRNA designed with a PBS of 37°C (8 nt). Our Tm-based pegRNA design produced a significant increase in GFP restoration (Supplementary Figure S8g,h). In addition, we designed a pegRNA with a predicted Tm of ∼37°C (9 nt PBS) for correction of *SBDS* IVS2 + 2T > C, a splice site mutation associated with almost all Shwachman-diamond syndrome cases (37) (Supplementary Figure S8e, i). This common mutation is believed to be derived via gene conversion from a neighboring pseudogene, *SBDSP1* (38,39). Therefore, we tested the SBDS IVS2 + 2T > C correction pegRNA at the *SBDSP1* site in HEK293T cells, which has an identical sequence with the *SBDS* IVS2 + 2T > C target site. We were able to achieve high editing rates up to 49.3% with PE2 and 73% with PE3 (Supplementary Figure S8j). Similarly, we designed a pegRNA with a predicted Tm of ∼37°C (8 nt PBS) for the HEK4 target site (HEK4 + 5G→T) (Supplementary Figure S8f). We were able to achieve 29.7% editing rates with PE2 (Supplementary Figure S8k).

We also evaluated the utility of this PBS design parameter for prime editing in zebrafish embryos. PE RNPs have been used successfully to install germline mutations in zebrafish embryos with modest editing rates (<10%) for the introduction of point mutations (21). We focused on a mutation that leads to vascular malformations (VMs). VMs are associated with somatic and germline activating mutations in the gene encoding the endothelial-specific Angiopoietin-1 receptor tyrosine kinase, TEK (40,41). The most common germline mutation is an autosomal-dominant p.R849W change that leads to weak activation of the receptor (41). In zebrafish Tek, the homologous residue is R841. Here, we designed a prime editing strategy to introduce the p.R841W mutation and a neighboring synonymous mutation into the zebrafish *tek* locus (Supplementary Figure S9a). We designed two pegRNAs that differ in the PBS length, one (7 nt) with a predicted Tm of 37°C and another one (6 nt) with a predicted *T*_m_ near 28.5°C, which is the incubation temperature for zebrafish embryos. We observed that both these *tek* pegRNAs when delivered as PE2 RNPs to zebrafish embryos were able to efficiently introduce the desired codon conversions at the target site with an overall precise editing rate of ∼20–26% (Supplementary Figure S9b). Additionally, we tested if utilizing a PE3 approach would increase the rate of precise edits at the *tek* locus. We translated the pegRNA:sgRNA ratios that were optimized for the PE3 system in mammalian cells to zebrafish embryos. We saw a modest increase (∼1.2-fold) in precise editing rates when employing the PE3 approach compared to PE2, where we achieved an overall precise editing rate of 26–33% (Supplementary Figure S9b). Thus, optimizing the PBS length based on the reaction temperature for genome editing provides efficient editing outcomes in mammalian cells and zebrafish embryos.

### Transient cold shock enhances prime editor activity

**Figure 3.**
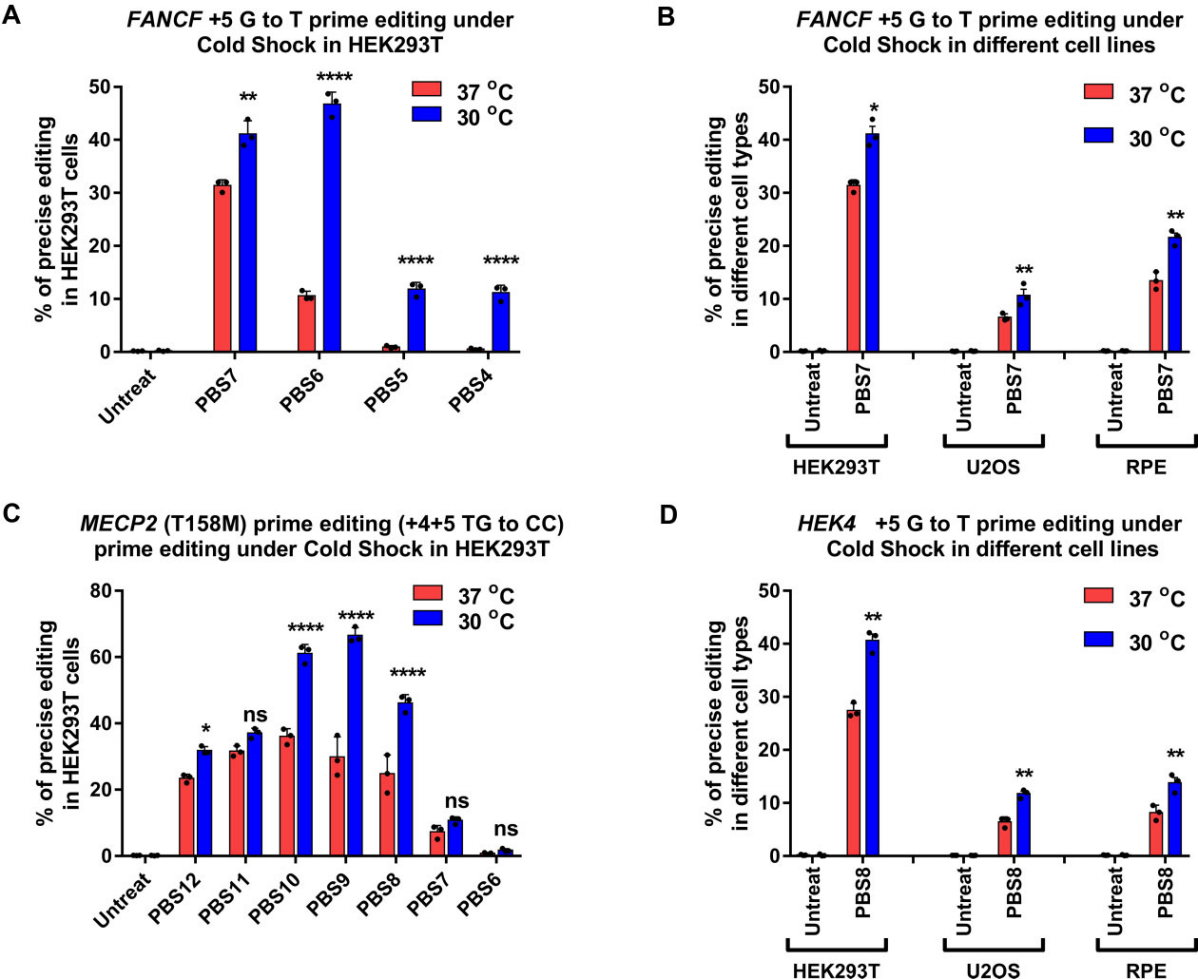
Cold shock enhances Prime editing rates in human cells. (A, C) RNP-mediated PE2 editing efficiency at the specified positions with varying pegRNA PBS length for (**A**) FANCF (+5 G to T) and (**C**) MECP2 (+4+5 TG to CC) loci in HEK293T cells. (B, D) RNP-mediated PE2 editing efficiency at the specified positions for (**B**) FANCF (+5 G to T) and (**D**) HEK4 (+5 G to T) loci in different cell lines (HEK293T, U2OS, RPE-1). 50 pmol PEmax protein and 100 pmol pegRNA were used for electroporation. Immediately after nucleofection, cells were incubated 3 days at 37°C or for cold shock, 12–16 h at 30°C followed by 2 days at 37°C. Editing efficiency reflects the frequency of sequencing reads that contain the intended precise edit among all amplicon deep sequencing reads. ‘Untreat’ indicates untreated cells. Values and error bars reflect mean ± s.d. of *n* = 3 independent biological replicates. Two-way ANOVA statistical analysis was used to determine the significance of prime editing at different temperature in different cell lines, ns indicates *P* > 0.05, ** indicates *P* ≤ 0.01, and **** indicates *P* ≤ 0.0001 (also see Supplementary table).

To further investigate if the PBS-target strand interaction is temperature dependent, we shifted the culture temperature of PE2 RNP treated cells post electroporation. We evaluated the prime editing efficiency of the FANCF pegRNA PBS panel in HEK293T, U2OS and RPE-1 cells at 30°C and 37°C. For the transient cold shock treatment, the cells were cultured at 30°C overnight for 12–16 hrs post nucleofection and then transferred to 37°C until the 72 h editing analysis point. We quantified the editing efficiency using targeted amplicon deep sequencing. Consistent with the importance of the reaction temperature on the PBS length for efficient prime editing, we observed an increase in prime editing activity for pegRNAs with shorter PBS lengths at 30°C relative to 37°C (Figure 3A). We also observed an unexpected increase in prime editing activity at 30°C for the optimal PBS length (7 nt) compared to the standard 37°C editing conditions. The observed increase in precise editing rates as a function of cold shock was independent of the cell type, where cold shock treatment increased the precise editing rates by 1.3 to 1.6 fold (Figure 3B). We observed a similar increase in prime editing rates at 30°C for pegRNAs targeting the HEK4 and MECP2 loci when using PE2 RNPs (Figure 3C,D). Thus, subjecting cells to a cold shock post electroporation can alter the prime editing activity as a function of PBS length and modestly enhance prime editing efficiency in a variety of cell types.

### Prime editing in patient-derived fibroblasts and human primary T cells

**Figure 4.**
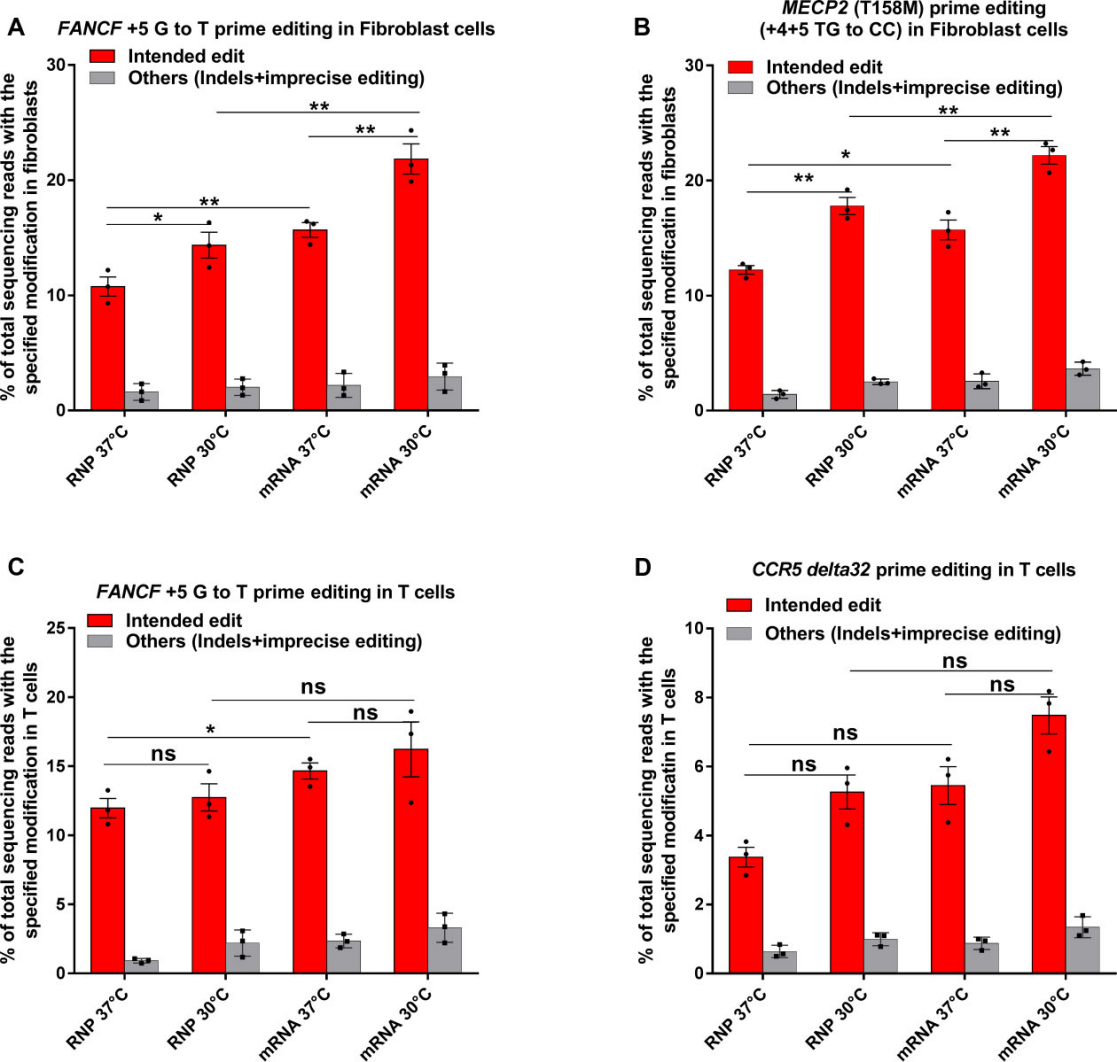
Prime editing efficiency in primary Fibroblasts and Primary T cells. (A, B) RNP and mRNA-mediated PE3 editing efficiencies at the specified positions for (**A**) *FANCF* (+5 G to T) and (**B**) *MECP2* (+4+5 TG to CC) in fibroblast cells at 30°C and 37°C. Editing efficiencies reflect the frequency of sequencing reads that contain the intended precise edit or others (indels and imprecise prime editing) among all sequencing reads. Bars and error bars represent mean ± s.d. (*n* = 3 biologically independent replicates). (**C**, **D**) RNP and mRNA-mediated PE3 editing efficiencies at the specified positions for *FANCF* (+5 G to T) and *CCR5* (+4+5 TG to CC) in Primary T cells at 30°C and 37°C. Editing efficiency reflects the frequencies of sequencing reads that contain the intended precise edit and others (indels and imprecise prime editing) among all sequencing reads. Bars and error bars represent mean ± s.d. (*n* = 3 biologically independent replicates). One-way ANOVA statistical analysis was used to determine the significance of precise prime editing at different temperatures, ns indicates *P* > 0.05, * indicates *P* ≤ 0.05, and ** indicates *P* ≤ 0.01 (also see Supplementary table).

The goal for improving prime editing systems is the modification of primary cells. Consequently, we repeated the experiments analyzing the impact of PBS length and cold shock on prime editing outcomes in patient derived fibroblasts. We tested a panel of pegRNAs with different PBS lengths at the FANCF and MECP2 loci in fibroblast cells and observed that shorter PBS lengths were preferred and that an increase in prime editing rates was obtained when the cells were subjected to cold shock treatment post PEmax mRNA electroporation (Supplementary Figure S10). Motivated by these results, we tested prime editing in different formats in a Rett syndrome patient-derived fibroblast line that carries the T158M mutation, and in primary human T cells. In the Rett fibroblast line, we tested PE3 RNP or PE3 mRNA delivery targeting the FANCF (+5 G→T) and MECP2 T158M sites employing a pegRNA with a 7 nt PBS for FANCF or a 10 nt PBS for T158M. We observed 10.8% and 15.7% +5 G→T edits at the FANCF target site with RNP and mRNA respectively and 12.2% and 15.9% correction of the mutant allele at the MECP2 target site with RNP and mRNA, respectively (Figure 4A, B). A transient cold shock treatment of these cells following electroporation further increased the prime editing efficiency by ∼1.5-fold at FANCF and MECP2 T158M for both PE3 RNP and PE3 mRNA delivery. In primary human T cells, we tested PE3 RNP or mRNA delivery targeting the FANCF (+5 G→T) site evaluating editing at both 37°C and with cold shock at 30°C. We observed 11.3% and 14.2% precise editing at 37°C with PE3 RNP and PE3 mRNA respectively, which increased ∼1.2-fold with a cold shock treatment (Figure 4C). We additionally designed a pegRNA to introduce the CCR5delta32 mutation into T cells, which is associated with HIV resistance (42). Using the MELTING 5 program (35), the optimal PBS length calculated for this pegRNA was 10 nt. Using a pegRNA with a 10 nt PBS with PE3 RNP or mRNA delivery by electroporation, we observed 3.4% and 5.1% rate of delta32 deletion with PE3 RNP and PE3 mRNA respectively when the T cells were grown at 37°C, and ∼1.4 fold increase in editing rates with a cold shock treatment (Figure 4D).

## DISCUSSION

In this study, we have evaluated factors that influence the efficiency of prime editing systems *in vitro* and in cell culture when employing prime editor protein – pegRNA (RNP) complexes. While investigating features of the pegRNA that influence Cas9 DNA cleavage rates *in vitro*, we found that the activity of the Cas9 nuclease protein loaded with a pegRNA was negligible when employing a PBS length (≥10 nt) that is optimal for editing with plasmid-expressed prime editor components. *In vitro* this ‘auto-inhibition’ associated with the pegRNA prevents SpCas9 nuclease from cleaving a DNA target site. The interaction between the PBS and spacer within the pegRNA has been previously described to reduce SpCas9 editing activity in cells (7). We found that this auto-inhibition is dependent on the length of the PBS region, such that utilizing shorter PBS lengths (7–8 nt) partially restores Cas9 DNA cleavage activity. This auto-inhibition for SpCas9 DNA cleavage can be relieved by introducing a DNA or RNA oligonucleotide that is complementary to the PBS-RTT region of the pegRNA, which implicates Watson-Crick pairing between the PBS and spacer region as being responsible for the inhibition of SpCas9 nuclease activity.

Interestingly, prior studies have examined the optimal PBS lengths in mammalian cells using plasmid-based (1) or lentivial-based (29) expression systems of the prime editor and pegRNA, where the pegRNA is expressed using a strong RNA polymerase III (U6) promoter. These studies have come to a common conclusion that a PBS length of ∼13 nt is optimal when the PBS base composition is between 50 and 60% G–C nucleotides. We have reproduced these results using plasmid expression systems interrogating the impact of PBS length on precise prime editing outcomes in transformed cell lines. However, our experiments using prime editor RNPs complexed with chemically synthesized, end-protected pegRNAs (three terminal phosphorothioates and 2’O-methyl groups at each end) delivered to cells by electroporation revealed that shorter PBS lengths have improved rates of precise genome editing presumably due to less auto-inhibitory interaction between the PBS and spacer. These PBS length results are consistent across multiple different target sites and when delivered to different cell types. Similar PBS length dependence is observed for prime editor mRNA delivery with chemically protected pegRNAs suggesting that reducing the complementarity between the PBS and spacer regions in end-protected pegRNAs increases prime editing efficiency. Prior studies looking at pegRNA designs have not directly addressed the complementarity between the PBS and spacer region. However, one study described introducing same-sense mutations in the RTT region that included the first three bases next to the PBS to increase prime editing efficiency (9). These three bases of the RTT share complementarity with the spacer sequence (Supplementary Figure S1b). Consequently, introducing synonymous mutations at these positions would reduce auto-inhibition and would be expected to increase the prime editing activity. This also explains why higher editing rates are observed for small deletions or insertions (that change or delete the bases between the nicking site and the PAM) when compared to point mutations, a trend that is apparent in studies performed by other groups (1,21).

The difference in PBS length requirement between plasmid expression systems and PE RNPs loaded with synthetic, end-protected pegRNAs appears to originate from the susceptibility of transcriptionally produced pegRNAs to 3’ end degradation (8). By small RNA sequencing, we observe that while the majority of the transcriptionally produced pegRNAs are full length, the pegRNA species that are bound by the prime editor protein are enriched for 3’ truncated species. The preferential binding of 3’ truncated pegRNA species to the prime editor protein is consistent with our *in vitro* competition assay between pegRNA and sgRNA loading onto Cas9. Since both the shorter and longer PBS pegRNAs are undergoing 3’ end processing (8), we speculate that the longer PBS length dependence for transcriptionally produced pegRNAs potentially provides these pegRNAs with a greater distribution of PBS lengths in the sweet spot of prime editing activity for complexing with the prime editor protein. In support of this theory, we show that plasmid delivery of epegRNAs that have pseudoknot structures at their 3’ end to reduce the rate of exonuclease degradation (8), produce higher precise prime editing rates with shorter PBS lengths. We also observed by small RNA sequencing that the epegRNA with a shorter PBS length had lower amounts of truncated species bound to the protein compared to the epegRNA with the longer PBS (Supplementary Figure S6). The driving force behind this difference in the distribution of loaded, truncated epegRNAs is unclear. We speculate that it may be a function of the level of PBS-spacer auto-inhibition, which is a function of PBS length. So, an epegRNA with a longer PBS might have stronger auto-inhibition interaction and therefore less binding affinity to the PE protein, which leads to loading of more truncated species. It could also be due to the larger fraction of truncated species that are observed in the bulk population, which will thereby partition based on their thermodynamic binding preference.

Computational analysis of the melting temperature (*T*_m_) of the PBS (RNA)–nicked spacer (DNA) interaction for the various target sites we tested indicates that the optimal *T*_m_ is ∼37°C for maximum editing rates in cell culture. Remarkably, this is the temperature that mammalian cells are incubated at for growth during genome editing. The Li and Gao laboratories have also used Tm as a metric for designing optimal PBS lengths for plant pegRNAs. They describe an optimal Tm of the PBS to be ∼30°C (10), which they calculated based on a simple formula of base composition [4°C*(each G or C nt) + 2°C*(each A or T nt)], as opposed to a thermodynamic analysis based on nearest-neighbor sequence composition (35). Consequently, their optimal PBS length is ∼10 nt for plant pegRNAs, which are expressed via plasmids and have no 3’ end protection. Based on our observation in mammalian systems, we anticipate that the optimal PBS length in plants when employing epegRNAs or synthetic end-protected pegRNAs would be shorter than 10 nt with the optimal Tm closer to their incubation temperature for plant protoplasts when employing a nearest-neighbor calculation of *T*_m_.

We also show that a cold shock treatment to the cells post PE RNP nucleofection significantly increases prime editing rates across multiple loci, different cell types, and different delivery methods (mRNA and RNP) (Supplementary Figure S11) A similar phenomenon was observed for zinc finger nuclease editing in cell culture, where the increase in editing activity due to cold shock was ascribed in part to increased nuclease stability at lower temperatures (43). Our analysis of editing dependence on PBS length as a function of temperature indicates that as the cell culture temperature is reduced, shorter PBS lengths are tolerated, or even preferred. Thus, some aspects of the PBS-spacer interaction may serve as a rate-limiting step in the editing reaction leading to desired prime editing outcomes, but the mechanism underlying the increased prime editing rates at lower temperature remains unclear. It is possible that growing the cells temporarily at 30°C might facilitate improved annealing of the PBS with the target DNA strand. Cold shock also slows cellular metabolism leading to slower progression through the cell cycle, which may stabilize important repair intermediates or provide more time for precise repair before DNA replication impacts DNA repair outcomes (44,45).

Finally, we show that the delivery of PE RNP complexes or PE mRNAs with pegRNAs that have a shorter PBS can achieve efficient editing in zebrafish embryos, patient-derived fibroblasts and primary T cells. Notably, prior prime editing rates in zebrafish embryos with PE RNPs for encoding base substitutions were below 10% and prime editing in primary T cells with PE RNP were typically 1.5–7.5% (21). With optimization of the prime editor protein and pegRNA components, we have substantially improved precise editing rates, achieving 30% precise editing for a base substitution in zebrafish and ∼15% in T cells. We believe that both PE mRNA and PE RNP modes of delivery will benefit from the PBS design parameters described within this study with the calculation of the Tm serving to define the optimal PBS length for pegRNAs. These principles should prove valuable for increasing the efficiency of engineering cell lines, plants and animals through prime editing. Moreover, given that many potential *in vivo* and *ex vivo* therapeutic applications of prime editing will employ end-protected pegRNA species (either synthetic or epegRNA), we believe that employing an optimal PBS length within the pegRNA design will maximize desired genome editing outcomes. Additional pegRNA design strategies that would decrease the auto-inhibition between the spacer and PBS region of the pegRNA without compromising its ability to recognize the target strand or prime with the nicked non-target strand, should also increase desired prime editing outcomes.

## Supplementary Material

gkad456_Supplemental_Files

## Data Availability

Illumina Sequencing data have been submitted to the Sequence Read Archive. These datasets are available under BioProject Accession number PRJNA907921 (https://www.ncbi.nlm.nih.gov/bioproject/?term=PRJNA907921) (SRA: SRR23012416∼SRR23012421). The authors declare that all other data supporting the findings of this study are available within the paper and its Supplementary Information files or upon reasonable request. Backbone plasmids used for pegRNA and sgRNA cloning are available from Addgene (#122089). The PEmax protein expression vector will be deposited with Addgene.

## References

[1] Anzalone A.V. , Randolph P.B., Davis J.R., Sousa A.A., Koblan L.W., Levy J.M., Chen P.J., Wilson C., Newby G.A., Raguram A. et al. Search-and-replace genome editing without double-strand breaks or donor DNA. Nature. 2019; 576:149–157. 31634902 10.1038/s41586-019-1711-4 PMC6907074

[2] Rees H.A. , Liu D.R. Base editing: precision chemistry on the genome and transcriptome of living cells. Nat. Rev. Genet. 2018; 19:770–788. 30323312 10.1038/s41576-018-0059-1 PMC6535181

[3] Park S.J. , Jeong T.Y., Shin S.K., Yoon D.E., Lim S.Y., Kim S.P., Choi J., Lee H., Hong J.I., Ahn J. et al. Targeted mutagenesis in mouse cells and embryos using an enhanced prime editor. Genome Biol. 2021; 22:170. 34082781 10.1186/s13059-021-02389-w PMC8173820

[4] Liu P. , Liang S.Q., Zheng C., Mintzer E., Zhao Y.G., Ponnienselvan K., Mir A., Sontheimer E.J., Gao G., Flotte T.R. et al. Improved prime editors enable pathogenic allele correction and cancer modelling in adult mice. Nat. Commun. 2021; 12:2121. 33837189 10.1038/s41467-021-22295-w PMC8035190

[5] Song M. , Lim J.M., Min S., Oh J.S., Kim D.Y., Woo J.S., Nishimasu H., Cho S.R., Yoon S., Kim H.H. Generation of a more efficient prime editor 2 by addition of the Rad51 DNA-binding domain. Nat. Commun. 2021; 12:5617. 34556671 10.1038/s41467-021-25928-2 PMC8460726

[6] Chen P.J. , Hussmann J.A., Yan J., Knipping F., Ravisankar P., Chen P.F., Chen C., Nelson J.W., Newby G.A., Sahin M. et al. Enhanced prime editing systems by manipulating cellular determinants of editing outcomes. Cell. 2021; 184:5635–5652. 34653350 10.1016/j.cell.2021.09.018 PMC8584034

[7] Liu Y. , Yang G., Huang S., Li X., Wang X., Li G., Chi T., Chen Y., Huang X., Wang X. Enhancing prime editing by Csy4-mediated processing of pegRNA. Cell Res. 2021; 31:1134–1136. 34103663 10.1038/s41422-021-00520-x PMC8486859

[8] Nelson J.W. , Randolph P.B., Shen S.P., Everette K.A., Chen P.J., Anzalone A.V., An M., Newby G.A., Chen J.C., Hsu A. et al. Engineered pegRNAs improve prime editing efficiency. Nat. Biotechnol. 2022; 40:402–410. 34608327 10.1038/s41587-021-01039-7 PMC8930418

[9] Li X. , Zhou L., Gao B.Q., Li G., Wang X., Wang Y., Wei J., Han W., Wang Z., Li J. et al. Highly efficient prime editing by introducing same-sense mutations in pegRNA or stabilizing its structure. Nat. Commun. 2022; 13:1669. 35351879 10.1038/s41467-022-29339-9 PMC8964725

[10] Lin Q. , Jin S., Zong Y., Yu H., Zhu Z., Liu G., Kou L., Wang Y., Qiu J.L., Li J. et al. High-efficiency prime editing with optimized, paired pegRNAs in plants. Nat. Biotechnol. 2021; 39:923–927. 33767395 10.1038/s41587-021-00868-w

[11] Choi J. , Chen W., Suiter C.C., Lee C., Chardon F.M., Yang W., Leith A., Daza R.M., Martin B., Shendure J. Precise genomic deletions using paired prime editing. Nat. Biotechnol. 2022; 40:218–226. 34650269 10.1038/s41587-021-01025-z PMC8847327

[12] Anzalone A.V. , Gao X.D., Podracky C.J., Nelson A.T., Koblan L.W., Raguram A., Levy J.M., Mercer J.A.M., Liu D.R. Programmable deletion, replacement, integration and inversion of large DNA sequences with twin prime editing. Nat. Biotechnol. 2022; 40:731–740. 34887556 10.1038/s41587-021-01133-w PMC9117393

[13] Wang J. , He Z., Wang G., Zhang R., Duan J., Gao P., Lei X., Qiu H., Zhang C., Zhang Y. et al. Efficient targeted insertion of large DNA fragments without DNA donors. Nat. Methods. 2022; 19:331–340. 35228726 10.1038/s41592-022-01399-1

[14] Ferreira da Silva J. , Oliveira G.P., Arasa-Verge E.A., Kagiou C., Moretton A., Timelthaler G., Jiricny J., Loizou J.I. Prime editing efficiency and fidelity are enhanced in the absence of mismatch repair. Nat. Commun. 2022; 13:760. 35140211 10.1038/s41467-022-28442-1 PMC8828784

[15] Schene I.F. , Joore I.P., Oka R., Mokry M., van Vugt A.H.M., van Boxtel R., van der Doef H.P.J., van der Laan L.J.W., Verstegen M.M.A., van Hasselt P.M. et al. Prime editing for functional repair in patient-derived disease models. Nat. Commun. 2020; 11:5352. 33097693 10.1038/s41467-020-19136-7 PMC7584657

[16] Surun D. , Schneider A., Mircetic J., Neumann K., Lansing F., Paszkowski-Rogacz M., Hanchen V., Lee-Kirsch M.A., Buchholz F. Efficient generation and correction of mutations in human iPS cells utilizing mRNAs of CRISPR base editors and prime editors. Genes (Basel). 2020; 11:511. 32384610 10.3390/genes11050511 PMC7288465

[17] Adikusuma F. , Lushington C., Arudkumar J., Godahewa G.I., Chey Y.C.J., Gierus L., Piltz S., Geiger A., Jain Y., Reti D. et al. Optimized nickase- and nuclease-based prime editing in human and mouse cells. Nucleic Acids Res. 2021; 49:10785–10795. 34534334 10.1093/nar/gkab792 PMC8501948

[18] Zhi S. , Chen Y., Wu G., Wen J., Wu J., Liu Q., Li Y., Kang R., Hu S., Wang J. et al. Dual-AAV delivering split prime editor system for in vivo genome editing. Mol. Ther. 2022; 30:283–294. 34298129 10.1016/j.ymthe.2021.07.011 PMC8753371

[19] Gao Z. , Ravendran S., Mikkelsen N.S., Haldrup J., Cai H., Ding X., Paludan S.R., Thomsen M.K., Mikkelsen J.G., Bak R.O. A truncated reverse transcriptase enhances prime editing by split AAV vectors. Mol. Ther. 2022; 30:2942–2951. 35808824 10.1016/j.ymthe.2022.07.001 PMC9481986

[20] Zheng C. , Liang S.Q., Liu B., Liu P., Kwan S.Y., Wolfe S.A., Xue W. A flexible split prime editor using truncated reverse transcriptase improves dual-AAV delivery in mouse liver. Mol. Ther. 2022; 30:1343–1351. 34998953 10.1016/j.ymthe.2022.01.005 PMC8899602

[21] Petri K. , Zhang W., Ma J., Schmidts A., Lee H., Horng J.E., Kim D.Y., Kurt I.C., Clement K., Hsu J.Y. et al. CRISPR prime editing with ribonucleoprotein complexes in zebrafish and primary human cells. Nat. Biotechnol. 2022; 40:189–193. 33927418 10.1038/s41587-021-00901-y PMC8553808

[22] Li H. , Busquets O., Verma Y., Syed K.M., Kutnowski N., Pangilinan G.R., Gilbert L.A., Bateup H.S., Rio D.C., Hockemeyer D. et al. Highly efficient generation of isogenic pluripotent stem cell models using prime editing. Elife. 2022; 11:e79208. 36069759 10.7554/eLife.79208 PMC9584603

[23] Kankia B.I. , Marky L.A. The Journal of Physical Chemistry B. 1999; 103:8759–8767.

[24] Pulikkan J.A. , Hegde M., Ahmad H.M., Belaghzal H., Illendula A., Yu J., O’Hagan K., Ou J., Muller-Tidow C., Wolfe S.A. et al. CBFbeta-SMMHC inhibition triggers apoptosis by disrupting MYC chromatin dynamics in acute myeloid leukemia. Cell. 2018; 174:172–186. 29958106 10.1016/j.cell.2018.05.048 PMC6211564

[25] Zou H. , Jakovlic I., Chen R., Zhang D., Zhang J., Li W.X., Wang G.T. The complete mitochondrial genome of parasitic nematode Camallanus cotti: extreme discontinuity in the rate of mitogenomic architecture evolution within the Chromadorea class. BMC Genomics (Electronic Resource). 2017; 18:840. 10.1186/s12864-017-4237-x PMC5669012 29096600

[26] Liu B. , Dong X., Cheng H., Zheng C., Chen Z., Rodriguez T.C., Liang S.Q., Xue W., Sontheimer E.J. A split prime editor with untethered reverse transcriptase and circular RNA template. Nat. Biotechnol. 2022; 40:1388–1393. 35379962 10.1038/s41587-022-01255-9

[27] Wu Y. , Zeng J., Roscoe B.P., Liu P., Yao Q., Lazzarotto C.R., Clement K., Cole M.A., Luk K., Baricordi C. et al. Highly efficient therapeutic gene editing of human hematopoietic stem cells. Nat. Med. 2019; 25:776–783. 30911135 10.1038/s41591-019-0401-y PMC6512986

[28] Clement K. , Rees H., Canver M.C., Gehrke J.M., Farouni R., Hsu J.Y., Cole M.A., Liu D.R., Joung J.K., Bauer D.E. et al. CRISPResso2 provides accurate and rapid genome editing sequence analysis. Nat. Biotechnol. 2019; 37:224–226. 30809026 10.1038/s41587-019-0032-3 PMC6533916

[29] Kim H.K. , Yu G., Park J., Min S., Lee S., Yoon S., Kim H.H. Predicting the efficiency of prime editing guide RNAs in human cells. Nat. Biotechnol. 2021; 39:198–206. 32958957 10.1038/s41587-020-0677-y

[30] Philippe C. , Villard L., De Roux N., Raynaud M., Bonnefond J.P., Pasquier L., Lesca G., Mancini J., Jonveaux P., Moncla A. et al. Spectrum and distribution of MECP2 mutations in 424 Rett syndrome patients: a molecular update. Eur. J. Med. Genet. 2006; 49:9–18. 16473305 10.1016/j.ejmg.2005.04.003

[31] Canver M.C. , Smith E.C., Sher F., Pinello L., Sanjana N.E., Shalem O., Chen D.D., Schupp P.G., Vinjamur D.S., Garcia S.P. et al. BCL11A enhancer dissection by Cas9-mediated in situ saturating mutagenesis. Nature. 2015; 527:192–197. 26375006 10.1038/nature15521 PMC4644101

[32] Sternberg S.H. , Redding S., Jinek M., Greene E.C., Doudna J.A. DNA interrogation by the CRISPR RNA-guided endonuclease Cas9. Nature. 2014; 507:62–67. 24476820 10.1038/nature13011 PMC4106473

[33] Trojan J. , Zeuzem S., Randolph A., Hemmerle C., Brieger A., Raedle J., Plotz G., Jiricny J., Marra G. Functional analysis of hMLH1 variants and HNPCC-related mutations using a human expression system. Gastroenterology. 2002; 122:211–219. 11781295 10.1053/gast.2002.30296

[34] Peng M. , Xie J., Ucher A., Stavnezer J., Cantor S.B. Crosstalk between BRCA-Fanconi anemia and mismatch repair pathways prevents MSH2-dependent aberrant DNA damage responses. EMBO J. 2014; 33:1698–1712. 24966277 10.15252/embj.201387530 PMC4194102

[35] Dumousseau M. , Rodriguez N., Juty N., Le Novere N. MELTING, a flexible platform to predict the melting temperatures of nucleic acids. BMC Bioinf. 2012; 13:101. 10.1186/1471-2105-13-101 PMC3733425 22591039

[36] Iyer S. , Mir A., Vega-Badillo J., Roscoe B.P., Ibraheim R., Zhu L.J., Lee J., Liu P., Luk K., Mintzer E. et al. Efficient homology-directed repair with circular single-stranded DNA donors. CRISPR J. 2022; 5:685–701. 36070530 10.1089/crispr.2022.0058 PMC9595650

[37] Woloszynek J.R. , Rothbaum R.J., Rawls A.S., Minx P.J., Wilson R.K., Mason P.J., Bessler M., Link D.C. Mutations of the SBDS gene are present in most patients with Shwachman-Diamond syndrome. Blood. 2004; 104:3588–3590. 15284109 10.1182/blood-2004-04-1516

[38] Boocock G.R. , Morrison J.A., Popovic M., Richards N., Ellis L., Durie P.R., Rommens J.M. Mutations in SBDS are associated with Shwachman-Diamond syndrome. Nat. Genet. 2003; 33:97–101. 12496757 10.1038/ng1062

[39] Nakashima E. , Mabuchi A., Makita Y., Masuno M., Ohashi H., Nishimura G., Ikegawa S. Novel SBDS mutations caused by gene conversion in Japanese patients with Shwachman-Diamond syndrome. Hum. Genet. 2004; 114:345–348. 14749921 10.1007/s00439-004-1081-2

[40] Vikkula M. , Boon L.M., Carraway K.L. 3rd, Calvert J.T., Diamonti A.J., Goumnerov B., Pasyk K.A., Marchuk D.A., Warman M.L., Cantley L.C. et al. Vascular dysmorphogenesis caused by an activating mutation in the receptor tyrosine kinase TIE2. Cell. 1996; 87:1181–1190. 8980225 10.1016/s0092-8674(00)81814-0

[41] Limaye N. , Wouters V., Uebelhoer M., Tuominen M., Wirkkala R., Mulliken J.B., Eklund L., Boon L.M., Vikkula M. Somatic mutations in angiopoietin receptor gene TEK cause solitary and multiple sporadic venous malformations. Nat. Genet. 2009; 41:118–124. 19079259 10.1038/ng.272 PMC2670982

[42] Liu R. , Paxton W.A., Choe S., Ceradini D., Martin S.R., Horuk R., MacDonald M.E., Stuhlmann H., Koup R.A., Landau N.R. Homozygous defect in HIV-1 coreceptor accounts for resistance of some multiply-exposed individuals to HIV-1 infection. Cell. 1996; 86:367–377. 8756719 10.1016/s0092-8674(00)80110-5

[43] Doyon Y. , Choi V.M., Xia D.F., Vo T.D., Gregory P.D., Holmes M.C. Transient cold shock enhances zinc-finger nuclease-mediated gene disruption. Nat. Methods. 2010; 7:459–460. 20436476 10.1038/nmeth.1456

[44] Roobol A. , Carden M.J., Newsam R.J., Smales C.M. Biochemical insights into the mechanisms central to the response of mammalian cells to cold stress and subsequent rewarming. FEBS J. 2009; 276:286–302. 19054067 10.1111/j.1742-4658.2008.06781.x

[45] Maurissen T.L. , Woltjen K. Synergistic gene editing in human iPS cells via cell cycle and DNA repair modulation. Nat. Commun. 2020; 11:2876. 32513994 10.1038/s41467-020-16643-5 PMC7280248

[46] Liang S.-Q. , Liu P., Ponnienselvan K., Suresh S., Chen Z., Kramme C., Chatterjee P., Zhu L.J., Sontheimer E.J., Xue W. et al. Genome-wide profiling of prime editor off-target sites in vitro and in vivo using PE-tag. Nat. Methods. 2023; 10.1038/s41592-023-01859-2. PMC11708963 37156841

