# Supplementary material for: Reducing the inherent auto-inhibitory interaction within the pegRNA enhances prime editing efficiency
Source: Nucleic Acids Res. 2023 May 29;51(13):6966–80. doi: 10.1093/nar/gkad456 (PMC10359601; doi:10.1093/nar/gkad456)
Supplement: gkad456_Supplemental_Files [file gkad456_supplemental_files.zip › Final Revised- NAR - Supp data, protocol and note.pdf]

Supplementary figures

Sup Figure 1

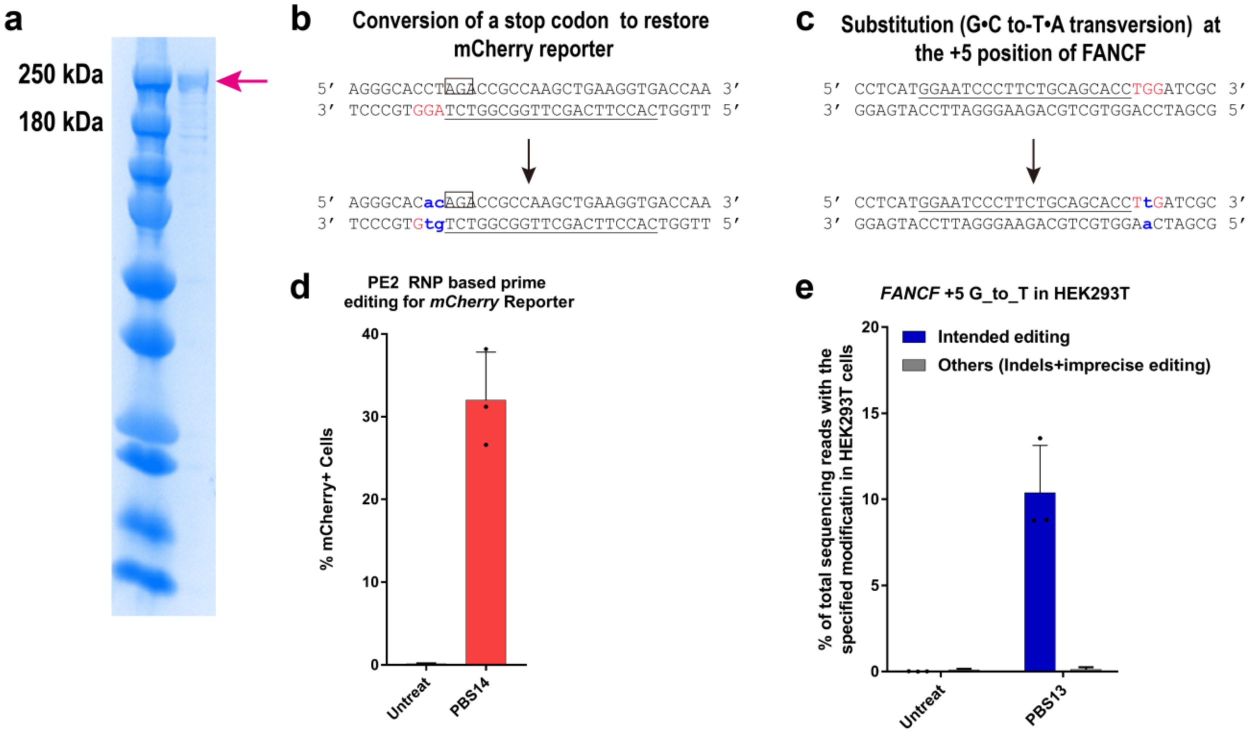

(**a**) SDS-PAGE Gel image showing purified C-terminally His-tagged PEmax protein. Magenta arrow indicates the desired protein product.

(**b**) Prime editing strategy for converting the stop codon to restore mCherry expression in the reporter cell line. The sequence in red denotes the PAM and the underlined sequence denotes the spacer region of the pegRNA. The boxed sequence denotes the stop codon that needs to be converted to a glutamine to restore sequence function. The nucleotides in blue are the edits incorporated to change the stop codon and the PAM sequence.

(**c**) Prime editing strategy to introduce G->T transversion mutation at the +5 position of the FANCF target site. The sequence in red denotes the PAM and the underlined sequence denotes the spacer region of the pegRNA. The nucleotide in blue denotes the edit incorporated at the +5 position.

(**d**) PE2 RNP based prime editing using pegRNA with 14 nt PBS for *mCherry* in HEK293T cells. 50 pmol PEmax protein and 100 pmol pegRNA were used for the electroporation. Frequency of mCherry positive cells was quantified by flow cytometry.

(**e**) PE2 RNP based prime editing using pegRNA with 13 nt PBS for base substitution (G•C to T•A transversion) at the +5 position of *FANCF* site. Editing efficiency reflects the frequency of sequencing reads that contain the intended prime edit or others (indels and imprecise prime editing) among all sequencing reads from amplicon deep sequencing. Values and error bars reflect mean ±s.d. of n=3 independent biological replicates.

Sup Figure 2

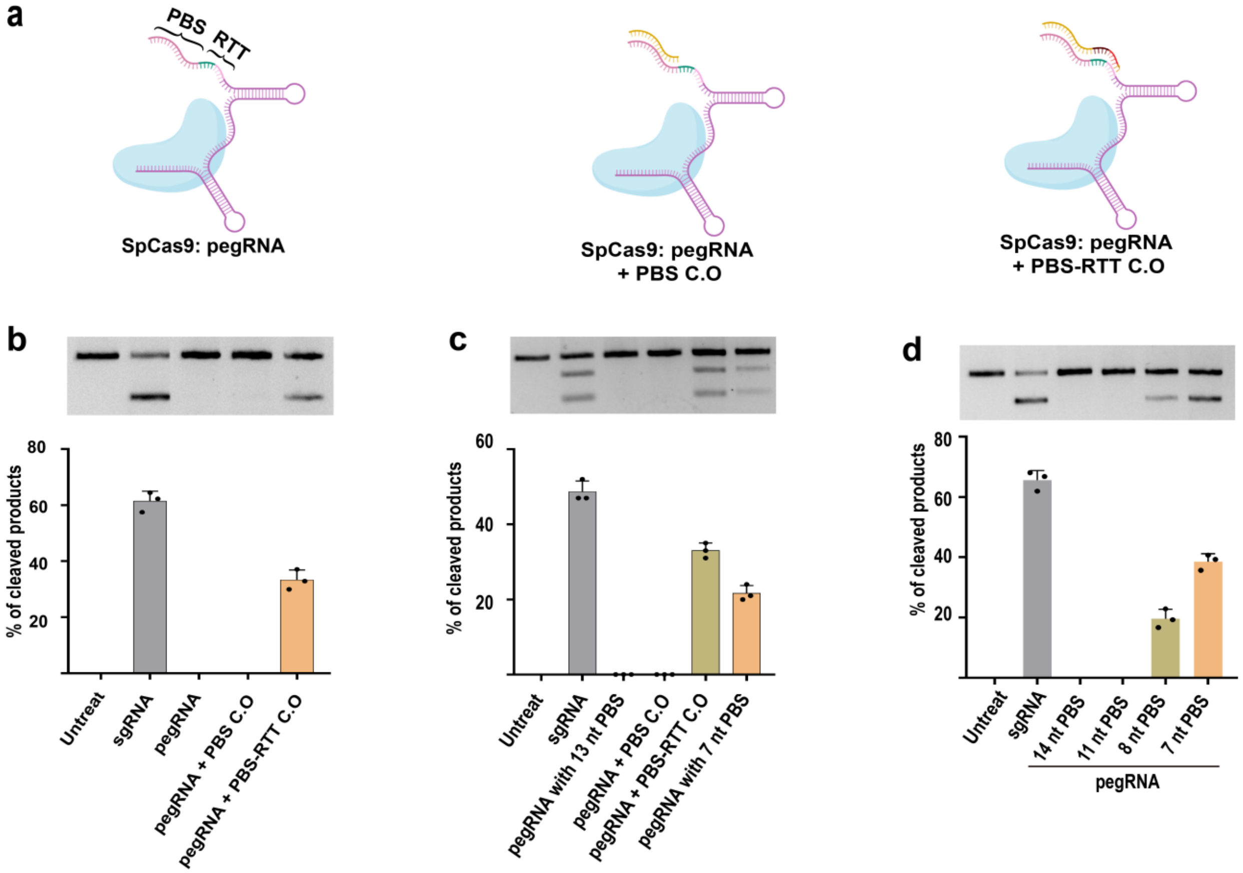

(**a**) Schematic showing the DNA competing oligonucleotides (C.Os) used for the *in vitro* cleavage assays. C.Os complementary to the PBS or the entire PBS-RTT region were used to relieve the auto-inhibitory interaction between the PBS<>spacer.

(**b**) *In vitro* cleavage data showing that mCherry pegRNA with a 14 nt PBS is inactive for Cas9 nuclease-based cleavage until a C.O complementary to the PBS-RTT is used to disrupt the PBS<>spacer interaction. 5 pmol of Cas9 was complexed with 10 pmol of pegRNA or sgRNA and 50 pmol of C.O complementary to the PBS or PBS+RTT was included where indicated. The RNP complex was incubated with 500 ng of target DNA for 20 minutes to carry out the cleavage reaction. Gel image is a representative outcome of one of three independent experiments. Values and error bars reflect mean ±s.d. of n=3 independent replicates.

(**c**) *In vitro* cleavage data showing that FANCF pegRNA is inactive for Cas9 nuclease based cleavage until a C.O complementary to the PBS-RTT is used to disrupt the PBS-spacer interaction. Reducing the PBS length to 7 nt results in a FANCF pegRNA that can program Cas9 to cleave the target site. 5 pmol of Cas9 protein was complexed with 10 pmol of pegRNA or sgRNA and 50 pmol of C.O complementary to the PBS or PBS+RTT was included where indicated. The RNP complex was incubated with 500 ng of target DNA for 20 minutes to carry out the cleavage reaction. Gel image is a representative outcome of one of three independent experiments. Values and error bars reflect mean ±s.d. of n=3 independent replicates.

(**d**) *In vitro* cleavage data showing that reducing the length of the PBS within the mCherry pegRNA increases the Cas9 nuclease cleavage rate of a cognate target site. 5 pmol of Cas9 protein was complexed with 10 pmol of pegRNA or sgRNA. The RNP complex was incubated with 500 ng of target DNA for 20 minutes to carry out the cleavage reaction. Gel image is a representative outcome of one of three independent experiments. Values and error bars reflect mean ±s.d. of n=3 independent replicates.

Sup Figure 3

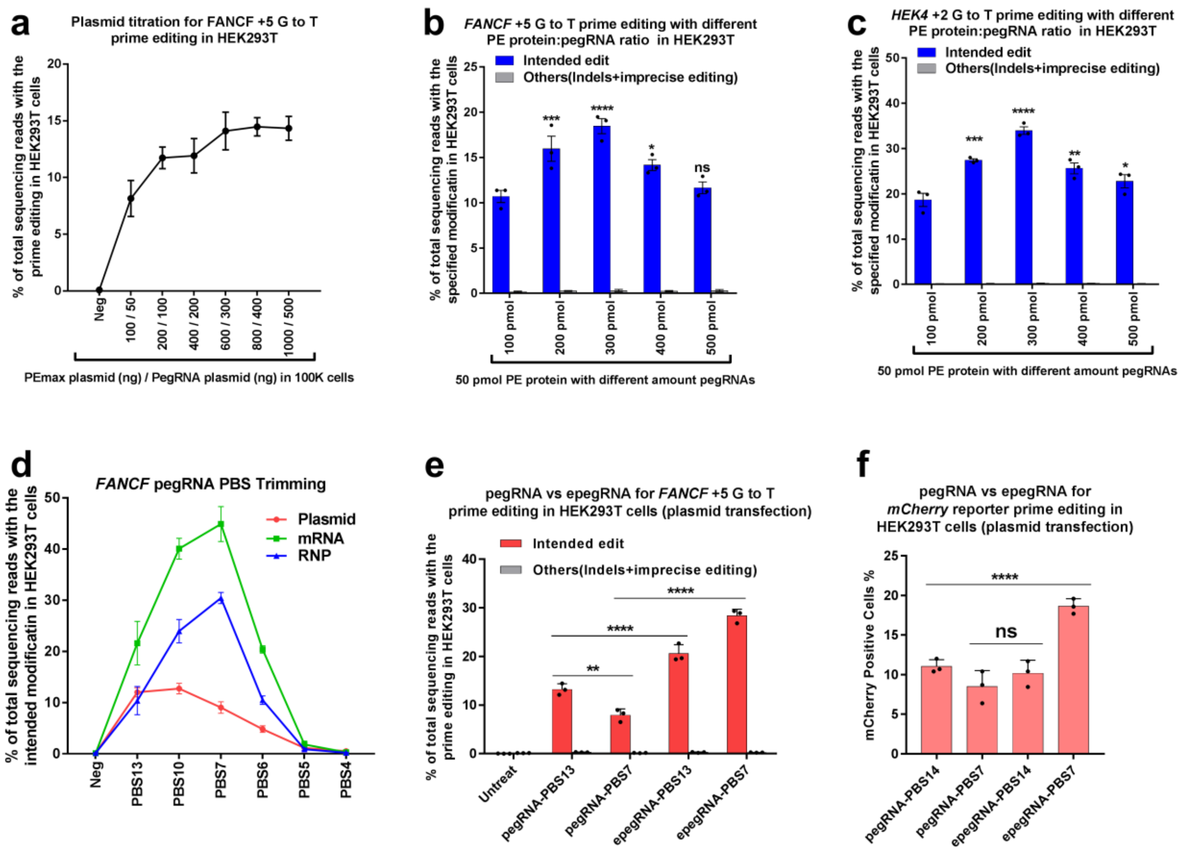

(**a**) Prime editing activities at *FANCF* (+5 G to T) titrating different amounts of expression plasmids for PEmax and pegRNA delivered by transient transfection to 100k HEK293T cells. The mass ratio of the PEmax-encoding plasmid and pegRNA-encoding plasmid were maintained at 2:1. Editing efficiency reflects the frequency of sequencing reads that contain the intended precise edit among all sequencing reads from amplicon deep sequencing. Values and error bars reflect mean ±s.d. of n=3 independent biological replicates.

(**b, c**) Prime editing activities at (**b**) *FANCF* (+5 G to T) and (**c**) *HEK4* (+5 G to T) using PEmax RNP programmed with the appropriate pegRNA delivered by electroporation to HEK293T cells. The molar ratio of PE protein:pegRNA delivered was varied between 1:2 and 1:10, maintaining the PE protein at 50 pmol. Editing efficiency reflects the frequency of sequencing reads that contain the intended edit or others (indels and imprecise prime editing) among all sequencing reads from amplicon deep sequencing. Values and error bars reflect mean ±s.d. of n=3 independent biological replicates. One-way ANOVA statistical analyses were used to compare the intended editing from different molar ratios of PE protein:pegRNA, 100 pmol pegRNA group was used as a control column for multiple comparisons. ns indicates P > 0.05, * indicates P ≤ 0.05, ** indicates P ≤ 0.01, and **** indicates P ≤ 0.0001(also see Supplementary table).

(**d**) Comparison of precise prime editing rates at *FANCF* (+5 G to T) between three different delivery platforms (transfection of expression plasmids encoding the prime editor and pegRNA, or electroporation of PE mRNA or RNP with synthetic pegRNAs) in HEK293T cells from experiments in Fig 1f-h.

(**e,f**) PE2 editing efficiencies in HEK293T cells using unmodified pegRNAs and epegRNAs containing the evopreQ1 pseudoknot with two different PBS lengths delivered via transient transfection (200 ng PEmax-encoding plasmid with 100 ng pegRNA or epegRNA-encoding plasmid). Editing efficiency reflects the frequency of sequencing reads from amplicon deep sequencing 72 hours following treatment that contain the intended edit or others (indels and imprecise prime editing) among all sequencing reads. Values and error bars reflect mean ±s.d. of n=3 independent biological replicates. One-way ANOVA statistical analysis were used for FANCF precise editing and mCherry reporter editing, respectively, ns indicates $P > 0.05$, ** indicates $P \leq 0.01$, and **** indicates $P \leq 0.0001$(also see Supplementary table).

Sup Figure 4

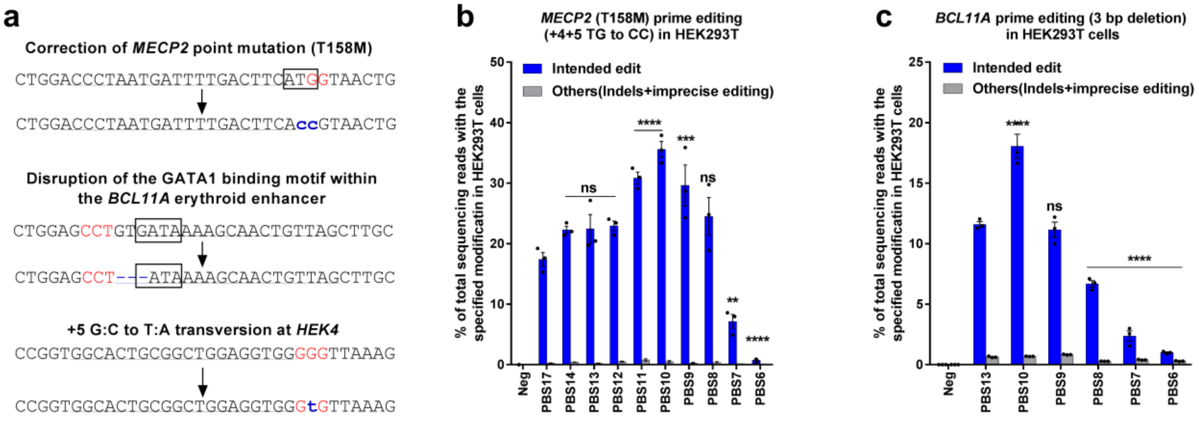

(**a**) Prime editing strategy to correct the T158M mutation in MECP2, disrupt the GATA1 binding motif of BCL11A erythroid enhancer or create +5 G->T mutation at the HEK4 target site. The sequence in red denotes the PAM and the underlined sequence denotes the spacer region of the pegRNA. The nucleotides in blue denote the edit incorporated. Dashes indicate deletions.

(**b,c**) Correction of the T158M mutation at MECP2 (**b**), or disruption of the GATA1 binding motif at BCL11A (**c**) in HEK293T cells with a panel of pegRNAs with different PBS lengths. 50 pmol PEmax protein and 200 pmol pegRNA (from IDT) were used for RNP electroporation. Editing efficiency reflects the frequency of sequencing reads that contain the intended prime editing or others (indels and imprecise prime editing) among all sequencing reads from amplicon deep sequencing. Values and error bars reflect mean ±s.d. of n=3 independent replicates. One-way ANOVA statistical analyses were used to compare the intended editing outcome rates from different pegRNAs, where the pegRNA with the longest PBS group (17 nt and 13 nt, respectively) was used as a control column for multiple comparisons. ns indicates P > 0.05, ** indicates P ≤ 0.01, *** indicates P ≤ 0.001, and **** indicates P ≤ 0.0001(also see Supplementary table).

Sup Figure 5

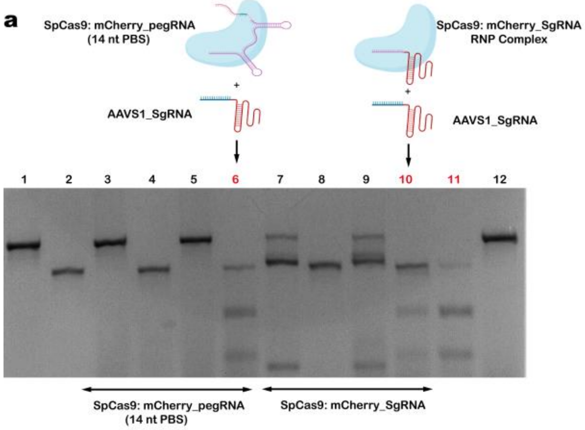

Lane	DNA target	Spcas9	Preloaded guide	Competition guide
1	mCherry	-	-	-
2	AAVS1	-	-	-
3	mCherry	+	mCherry pegRNA	-
4	AAVS1	+	mCherry pegRNA	-
5	mCherry	+	mCherry pegRNA	AAVS1 sgRNA
6	AAVS1	+	mCherry pegRNA	AAVS1 sgRNA
7	mCherry	+	mCherry sgRNA	-
8	AAVS1	+	mCherry sgRNA	-
9	mCherry	+	mCherry sgRNA	AAVS1 sgRNA
10	AAVS1	+	mCherry sgRNA	AAVS1 sgRNA
11	AAVS1	+	AAVS1 sgRNA	-
12	mCherry	+	AAVS1 sgRNA	-

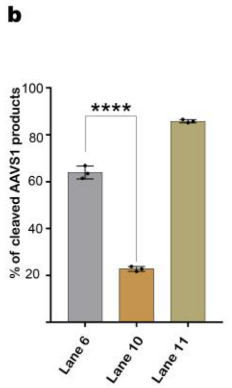

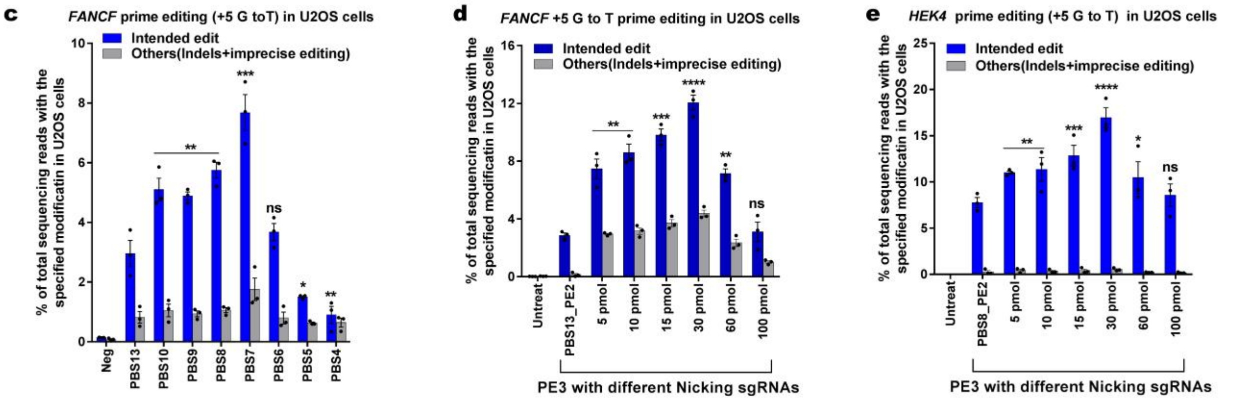

(**a**) *In vitro* competition based cleavage assay examining the relative binding efficiency of a pegRNA and sgRNA for Cas9. 500 ng of the PCR product was used for the DNA target, 5 pmol of Cas9 protein was complexed with 10 pmol of sgRNA or pegRNA for 20' followed by competition with 10 pmol of competing sgRNA wherever applicable (see table describing the contents of each lane). The resulting RNP complex was incubated with 500 ng of appropriate target DNA for 20 minutes to carry out the cleavage reaction. Lane 10 shows that when the mCherry sgRNA is loaded first on Cas9 and then competed with an sgRNA targeting the AAVS1 site, it marginally cleaves the AAVS1 PCR product. By contrast, in lane 6, when the mCherry pegRNA is loaded on Cas9 first and then competed with the AAVS1 sgRNA, it cleaves the AAVS1 PCR product to a greater extent. Gel image is a representative outcome of one of three independent experiments

(**b**) Quantification of the cleavage products from Lanes 6, 10 and 11 (table in **f**) indicating that AAVS1 sgRNA can effectively outcompete mCherry pegRNA for binding to Cas9 when compared to the mCherry sgRNA. Values and error bars reflect mean ±s.d. of n=3 independent replicates. Comparison of mean values was conducted with unpaired, two-tailed Student's t-test, **** indicates P ≤ 0.0001(also see Supplementary table).

(**c**) FANCF +5G->T edits in U2OS cells with a panel of pegRNAs with different PBS lengths. 50 pmol PEmax protein and 200 pmol pegRNA (from IDT) were used for RNP electroporation. Editing efficiency reflects the frequency of sequencing reads that contain the intended prime editing or others (indels and

imprecise prime editing) among all sequencing reads from amplicon deep sequencing. Values and error bars reflect mean ±s.d. of n=3 independent replicates. One-way ANOVA statistical analyses were used to compare the intended editing from different pegRNA, where the pegRNA with 13 nt PBS group was used as a control column for multiple comparisons. ns indicates P > 0.05, * indicates P ≤ 0.05, ** indicates P ≤ 0.01, *** indicates P ≤ 0.001, and **** indicates P ≤ 0.0001(also see Supplementary table).

(**d,e**) PE3 approach to introduce FANCF +5G->T edits and HEK4 +5G->T edits in U2OS cells with different concentrations of the nicking guide. The amount of PEmax protein (50 pmol) and pegRNA (200 pmol; IDT) was held constant while increasing the amount of nicking sgRNA (from IDT) delivered by electroporation. Editing efficiency reflects the frequency of sequencing reads that contain the intended prime editing or others (indels and imprecise prime editing) among all sequencing reads from amplicon deep sequencing. Values and error bars reflect mean ±s.d. of n=3 independent replicates. One-way ANOVA statistical analyses were used to compare the intended edit from different amounts of Nicking sgRNAs, PE2 group was used as a control column for multiple comparisons. ns indicates P > 0.05, * indicates P ≤ 0.05, ** indicates P ≤ 0.01, *** indicates P ≤ 0.001, and **** indicates P ≤ 0.0001(also see Supplementary table).

Sup Figure 6

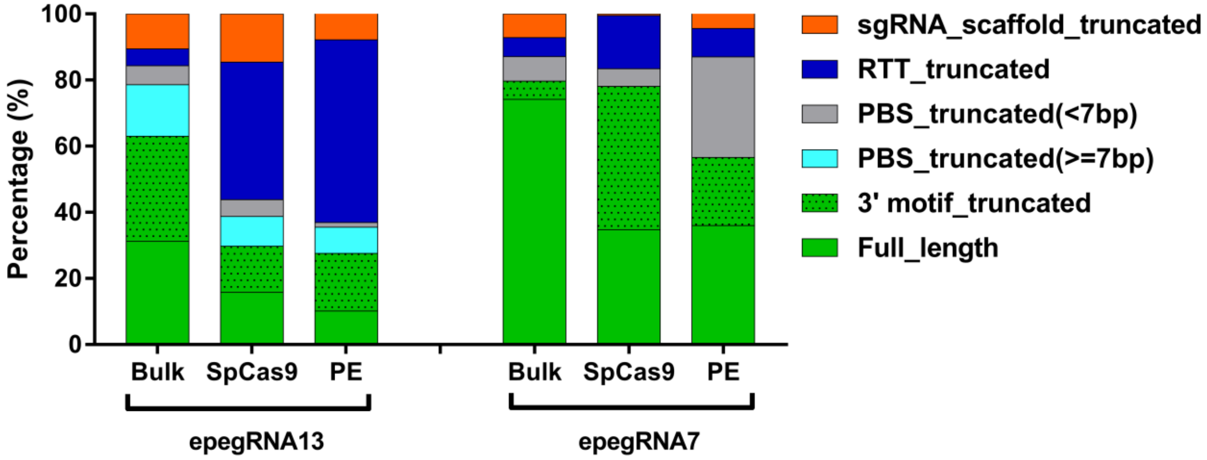

Bulk or effector-bound RNA species present from each treatment group (epegRNA with 13 nt or 7 nt PBS). Small RNAs were categorized into six species based on the length of 3' truncation: full-length epegRNA, epegRNA with truncated 3'motif, epegRNA with truncated but potentially functional PBS (≥ 7 nt remaining), epegRNA with truncated likely insufficient PBS (<7 nt), epegRNA with truncated RTT, and epegRNA with truncated sgRNA scaffold. Abundance of each RNA species was calculated based on UMIs incorporated into the 3' adaptor from the small RNA-seq library (see Supplementary Fig 7 for IGV plots).

Sup Figure 7

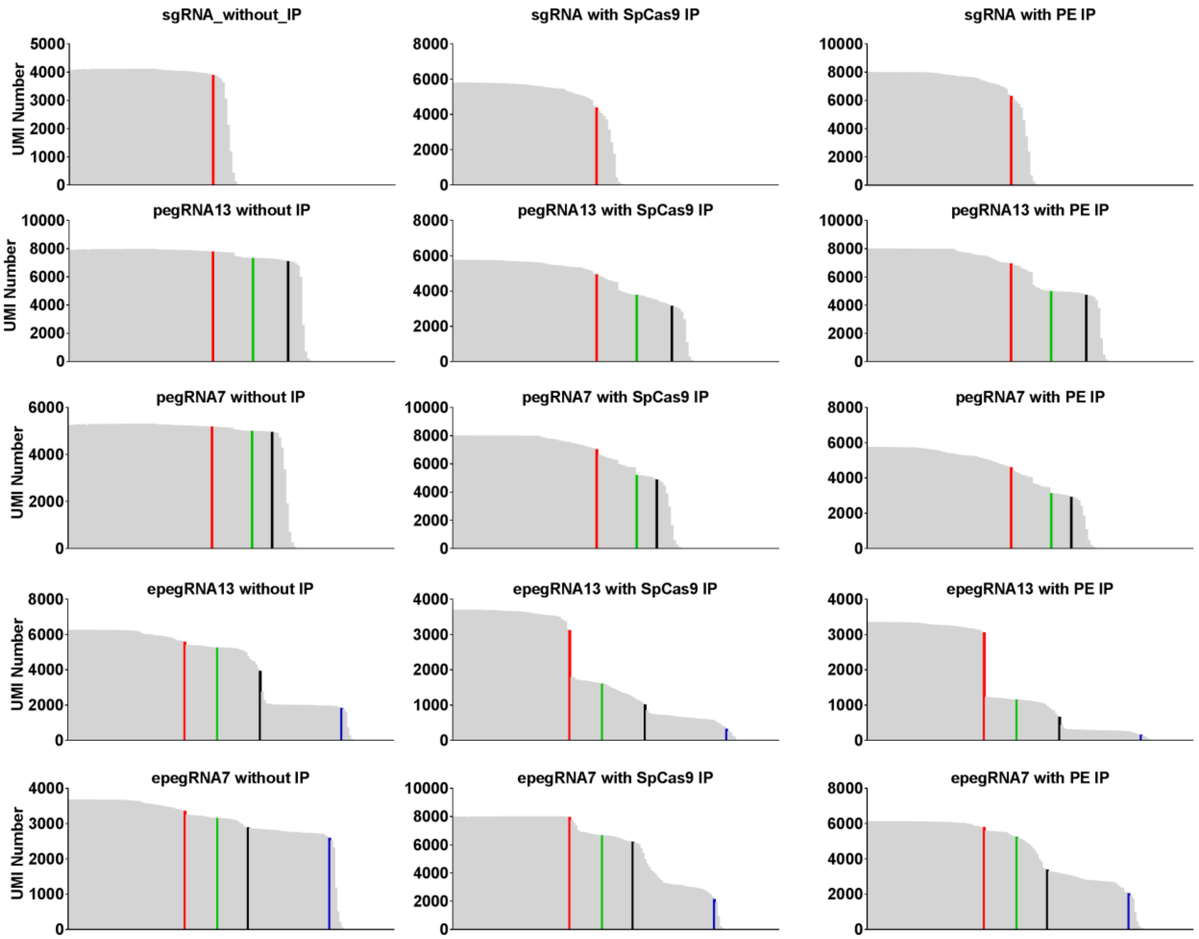

Coverage of deep sequencing reads across the ‘3 end of the sgRNA, pegRNA, and epegRNA delivered to HEK293T cells by plasmid expression vector through transient transfection (Figure 2 and Sup. Fig. 6). In each IGV trace the red line indicates the end of sgRNA scaffold, the green line indicates the end of PBS, the black line indicates the end of RTT, and the blue line indicates the end of 3’ pseudoknot motif in epegRNAs.

Sup Figure 8

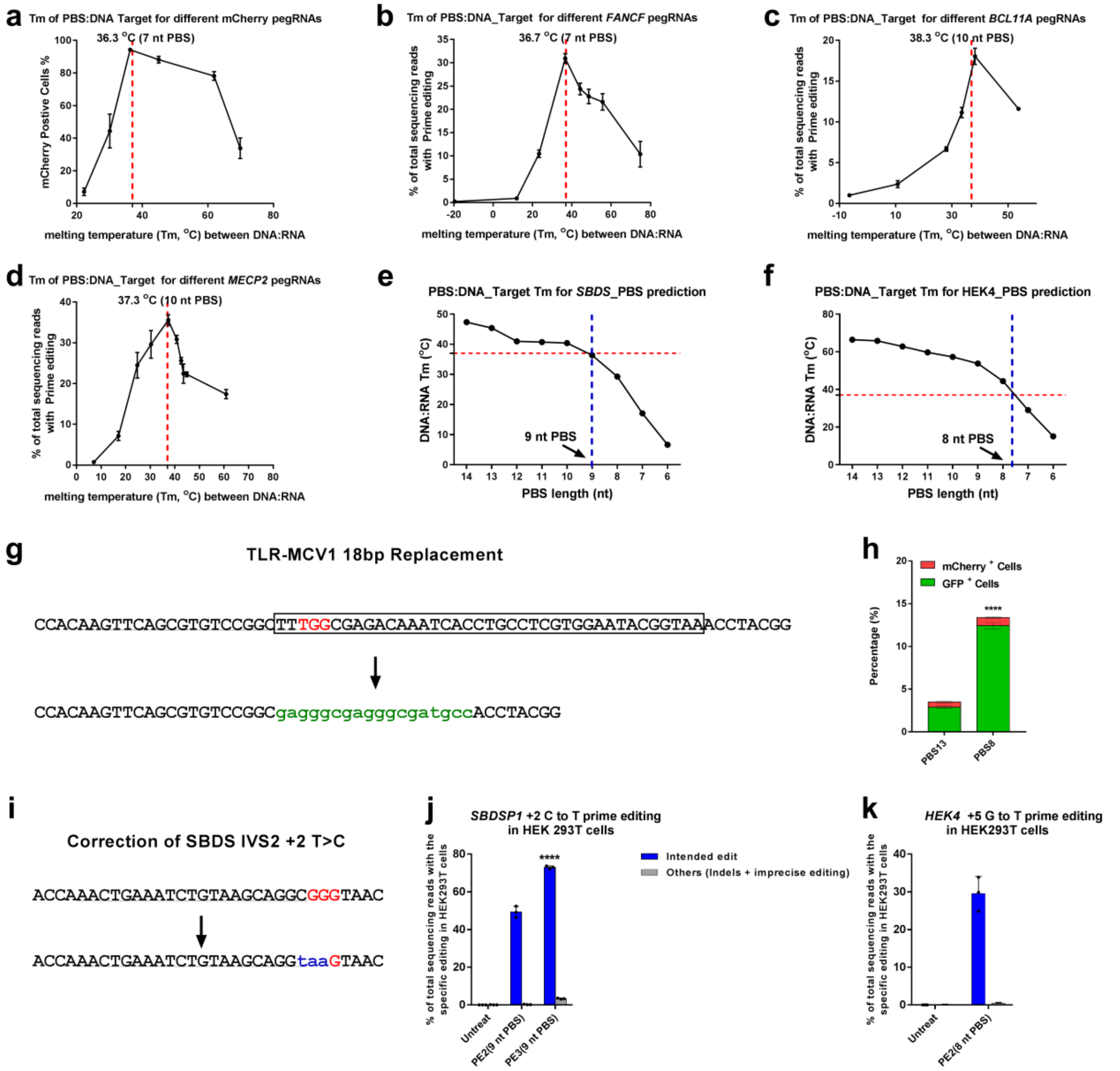

(**a,b,c,d**) Graphs indicating calculated Tm for the interaction between the nicked 3' DNA end produced by the prime editor and the PBS region of the pegRNA. Tms were calculated using the MELTING 5 software package for RNA-DNA hybrids(35). Tms are displayed as a function of the precise editing rate at four different loci (mCherry stop codon, FANCF +5G->T, BCL11A GATA1 disruption and correction of the T158M mutation at MECP2) in HEK293T cells. The highest editing rate is observed for a calculated Tm ~37°C.

(**e,f**) Graphs of MELTING 5 predicted Tms for pegRNAs with different PBS lengths targeting SBDS IVS2 +2T>C and +5G->T HEK4 for the interaction between the nicked 3' DNA end produced by the prime editor and the PBS region of the pegRNA. pegRNA designs targeting these loci were chosen based on

PBS lengths that have a predicted Tm ~37°C, 9 nt PBS for SBDS pegRNA, and 8 nt PBS for HEK4 pegRNA.

(**g**) 18bp replacement in the TLR-MCV1 HEK293T based reporter line. Sequence in red denotes the PAM and the sequence that is boxed is the sequence that is to be replaced with the sequence in green to result in a functional GFP product.

(**h**) The pegRNAs for TLR-MCV1 editing were designed based on conventional parameters (13 nt PBS, PBS13) and based on the Tm prediction (8 nt PBS, PBS8). GFP% denotes the amount of precise edits and mcherry% denotes indel rates.

(**i**) Correction of SBDS IVS2 +2T>C. +2 indicates the position of the transition mutation in intron 2 of SBDS, not the position of base conversion relative to the prime editor cleavage site. The sequence in red denotes the PAM and the underlined sequence denotes the spacer region of the pegRNA. The nucleotides in blue denote the edit incorporated.

(**j,k**) Editing rates at the (**j**) SBDSP1 and (**k**) HEK4 target site with pegRNAs that were designed based on the Tm predictions (panels e & f). For PE2, 50 pmol PEmax protein and 200 pmol pegRNA (from IDT) were used for RNP electroporation. For SBDSP1 PE3, 50 pmol PEmax protein, 200 pmol pegRNA and 15 pmol of nicking sgRNA were used for RNP electroporation. Editing efficiency reflects the frequency of sequencing reads that contain the intended prime editing or others (indels and imprecise prime editing) among all sequencing reads from amplicon deep sequencing. Values and error bars reflect mean ±s.d. of n=3 independent biological replicates. Comparison of PE2 and PE3 mediated intended edit for SBDSP1 was conducted with unpaired, two-tailed Student's t-test, **** indicates $P \leq 0.0001$(also see Supplementary table).

Sup Figure 9

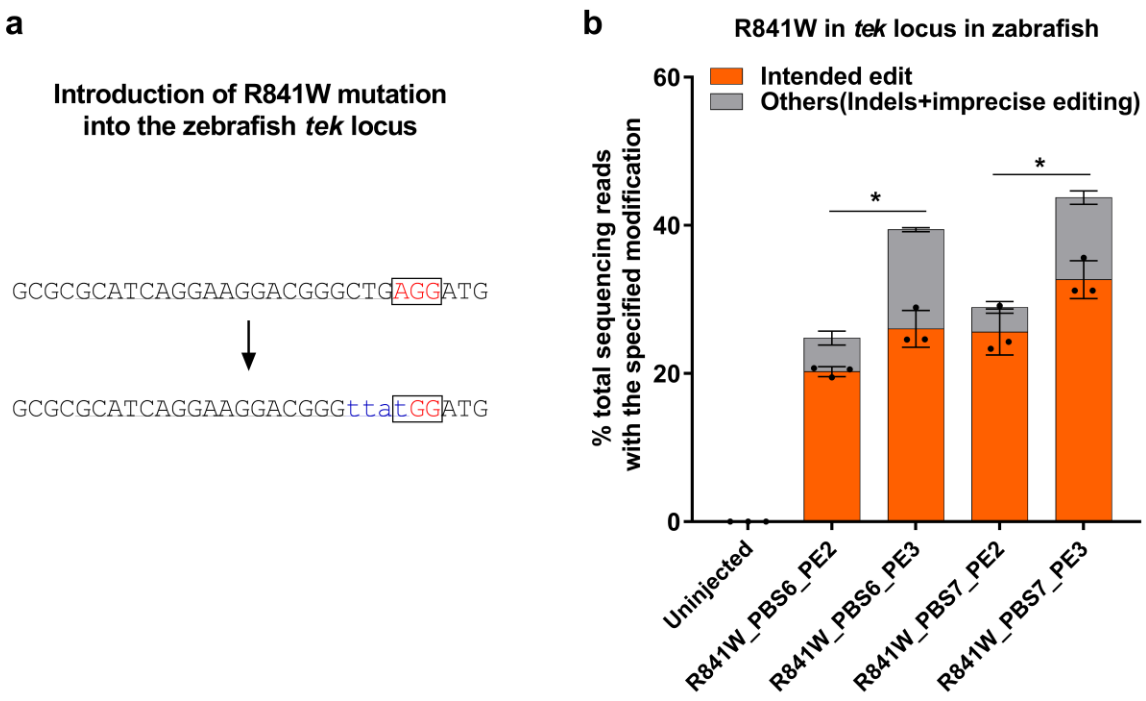

(**a**) Introduction of the Tek R841W mutation. The sequence in red denotes the PAM and the arginine codon to be changed. The lowercase nucleotides in blue denote the edit incorporated.

(**b**) Comparison between PE2 and PE3 approaches using pegRNAs with two different PBS lengths (6 and 7 nt) that introduce the R841W mutation at the *tek* locus in zebrafish. For PE2, 12 μM pegRNA (synthesized by IDT) and 6 μM PE protein were combined in nuclease-free water. For PE3 a nicking sgRNA (synthesized by IDT) was added to the PE2 complex at a 1 to 10 nicking sgRNA to pegRNA molar ratio. Editing efficiency reflects the frequency of sequencing reads that contain the intended precise edit or others (indels and imprecise prime editing) among all sequencing reads from amplicon deep sequencing. Values and error bars reflect mean ±s.d. of n=3 independent biological replicates. Comparisons of mean values of PE2- and PE3-mediated precise editing for the two different pegRNA were conducted with unpaired, two-tailed Student's t-test, * indicates P ≤ 0.05(also see Supplementary table).

Sup Figure 10

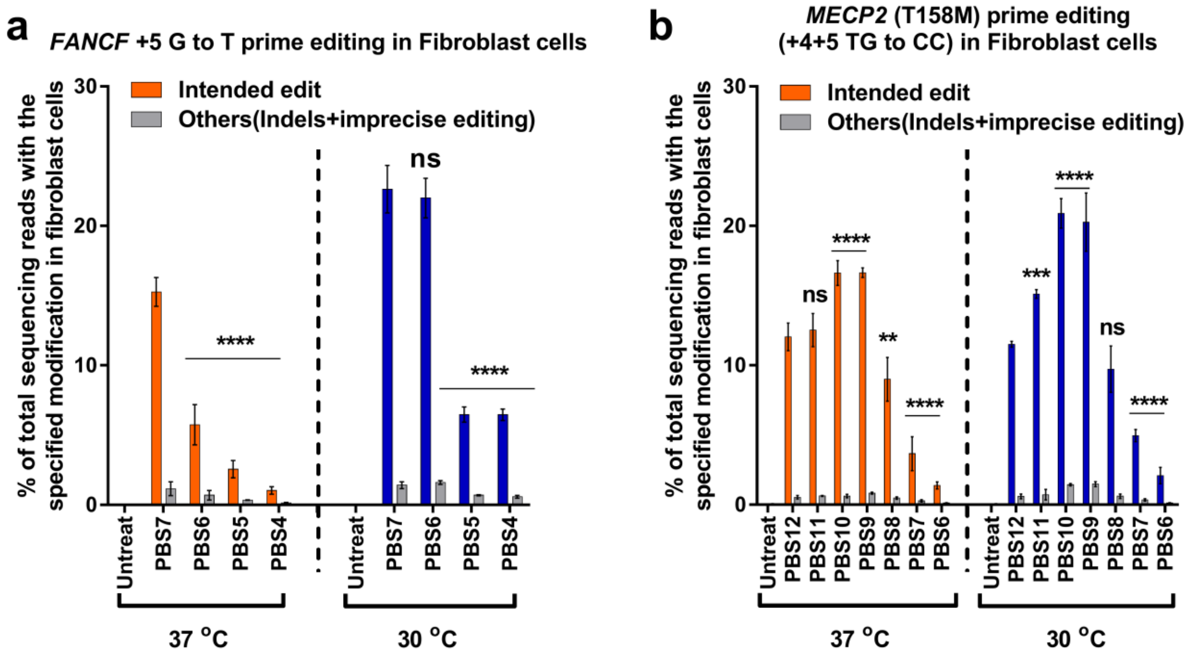

(**a, b**) RNP-mediated PE2 editing efficiency at the specified positions with varying pegRNA PBS lengths (length in nt indicated by PBS#), with or without transient cold shock for (**a**) FANCF (+5 G to T) and (**b**) MECP2 (+4+5 TG to CC) loci in patient derived fibroblasts.

Sup Figure 11

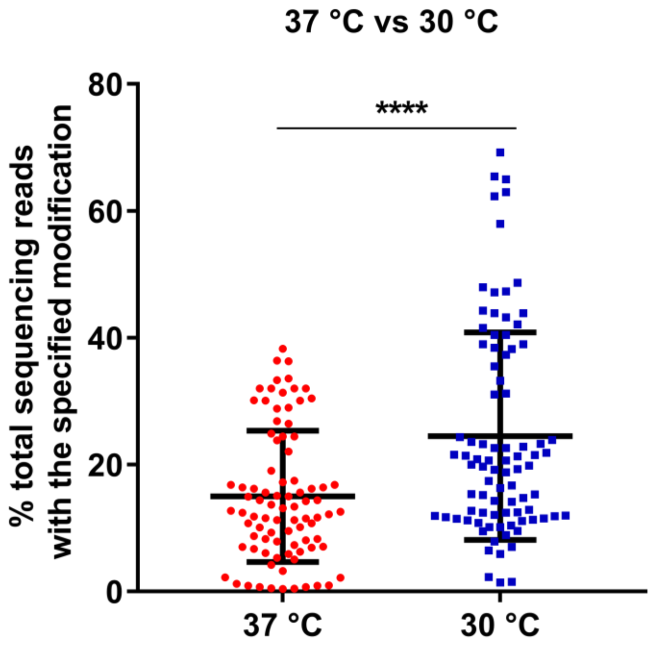

Comparison of prime editing efficiency at 30°C and 37°C across multiple loci, different cell types, and different delivery methods (mRNA and RNP), Comparison of mean values was conducted with paired, two-tailed Student's t-test; ** stands for P ≤ 0.01(also see Supplementary table).

Sup Figure 12

T7_tag-Cmyc_like_NLS-BPSV40_NLS-SpCas9 (E221K_N394K)-H840A-linkerHA_tag-linker-SV40_NLS-linker-M-MLV- SV40_NLS-Nucleoplasmin NLS-6His-Tag

```
MASMTGGQQMGRAAPAAKKKKLDGSVDKRTADGSEFESPKKKRKVDKKYSIGLDIGTNSVGWAVITDEYK
VPSKKFKVLGNTDRHSIKKNLIGALLFDSGETAEATRLKRTARRRYTRRKNRICYLQEIFSNEMAKVDDS
FFHRLEESFLVEEDKKHERHPIFGNIVDEVAYHEKYPTIYHLRKKLVDSTDKADLRLIYLALAHMIKFRG
HFLIEGDLNPDNSDVDKLFIQLVQTYNQLFEENPINASGVDAKAILSARLSKSRKLENLIAQLPGEKKNG
LFGNLIALSLGLTPNFKSNFDLAEDAKLQLSKDTYDDDLDNLLAQIGDQYADLFLAAKNLSDAILLSDIL
RVNTEITKAPLSASMIKRYDEHHQDLTLLKALVRQQLPEKYKEIFFDQSKNGYAGYIDGGASQEEFYKFI
KPILEKMDGTEELLVKLKREDLLRKQRTFDNGSIPHQIHLGELHAILRRQEDFYPFLKDNREKIEKILTF
RIPYYVGPLARGNSRFAWMTRKSEETITPWNFEEVVDKGASAQSFIERMTNFDKNLPNEKVLPKHSLLYE
YFTVYNELTKVKYVTEGMRKPAFLSGEQKKAIVDLLFKTNRKVTVKQLKEDYFKKIECFDSVEISGVEDR
FNASLGTYHDLLKIIKDKDFLDNEENEDILEDIVLTLTLFEDREMIEERLKTYAHLFDDKVMKQLKRRRY
TGWGRLSRKLINGIRDKQSGKTILDFLKSDGFANRNFMQLIHDDSLTFKEDIQKAQVSGQGDSLHEHIAN
LAGSPAIKKGILQTVKVVDELVKVMGRHKPENIVIEMARENQTTQKGQKNSRERMKRIEEGIKELGSQIL
KEHPVENTQLQNEKLYLYYLQNGRDMYVDQELDINRLSDYDVDAIVPQSFLKDDSIDNKVLTRSDKNRGK
SDNVPSEEVVKKMKNYWRQLLNAKLITQRKFDNLTKAERGGLSELDKAGFIKRQLVETRQITKHVAQILD
SRMNTKYDENDKLIREVKVITLKSKLVSDFRKDFQFYKVREINNYHHAHDAYLNAVVGTALIKKYPKLES
EFVYGDYKVYDVRKMIAKSEQEIGKATAKYFFYSNIMNFFKTEITLANGEIRKRPLIETNGETGEIVWDK
GRDFATVRKVLSMPQVNIVKKTEVQTGGFSKESILPKRNSDKLIARKKDWDPKKYGGFDSPTVAYSVLVV
AKVEKGKSKKLKSVKELLGITIMERSSFEKNPIDFLEAKGYKEVKKDLIIKLPKYSLFELENGRKRMLAS
AGELQKGNELALPSKYVNFLYLASHYEKLKGSPEDNEQKQLFVEQHKHYLDEIIEQISEFSKRVILADAN
LDKVLSAYNKHRDKPIREQAENIIHLFTLTNLGAPAAFKYFDTTIDRKRYTSTKEVLDATLIHQSITGLY
ETRIDLSQLGGDSGGSSGGSKRTAGSYPYDVPDYADGSEFESPKKKRKVSGGSSGGSTLNIEDEYRLHET
SKEPDVSLGSTWLSDFPQAWAETGGMGLAVRQAPLIIPLKATSTPVSIKQYPMSQEARLGIKPHIQRLLD
QGILVPCQSPWNTPLLPVKKPGTNDYRPVQDLREVNKRVEDIHPTVPNPYNLLSGLPPSHQWYTVLDLKD
AFFCLRLHPTSQPLFAFEWRDPEMGISGQLTWTRLPQGFKNSPTLFNEALHRDLADFRIQHPDLILLQYV
DDLLLAATSELDCQQGTRALLQTLGNLGYRASAKKAQICQKQVKYLGYLLKEGQRWLTEARKETVMGQPT
PKTPRQLREFLGKAGFCRLFIPGFAEMAAPLYPLTKPGTLFNWGPDQQKAYQEIKQALLTAPALGLPDLT
KPFELFVDEKQGYAKGVLTQKLGPWRRPVAYLSKKLDPVAAGWPPCLRMVAAIAVLTKDAGKLTMGQPLV
ILAPHAVEALVKQPPDRWLSNARMTHYQALLLDTDRVQFGPVVALNPATLLPLPEEGLQHNCLDILAEAH
GTRPDLTDQPLPDADHTWYTDGSSLLQEGQRKAGAAVTTETEVIWAKALPAGTSAQRAELIALTQALKMA
EGKKLNVYTDSRYAFATAHIHGEIYRRRGWLTSEGKEIKNKDEILALLKALFLPKRLSIIHCPGHQKGHS
AEARGNRMADQAARKAAITETPDTSTLLIENSSPSGGSTGGGPGGGAAAGSGSPKKKRKVGSGSKRPAAT
KKAGQAKKKKLEHHHHHH
```

Protein sequence of PEmax used for bacterial expression and purification.

Sup Figure 13

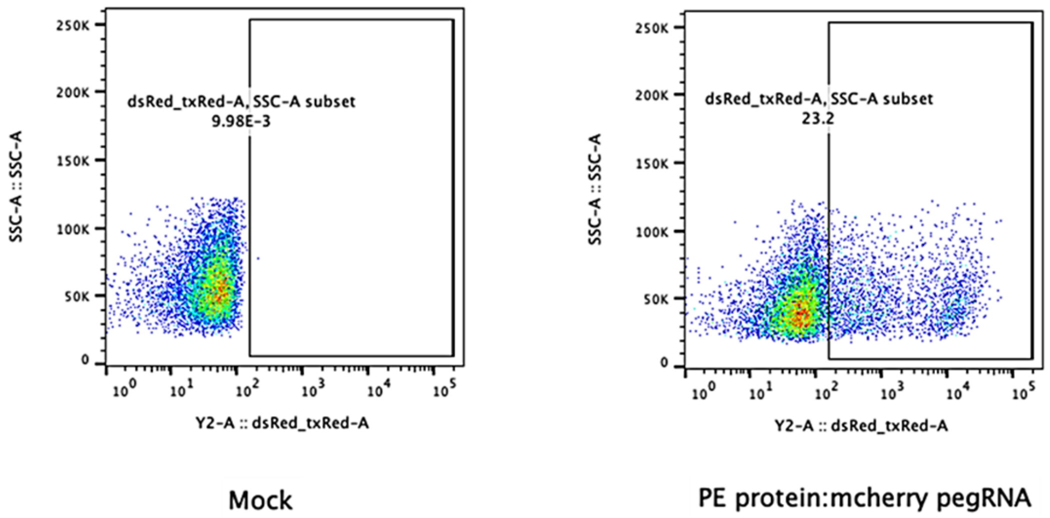

Gating strategy for the mCherry reporter cell line.

Supplementary Protocol

Small RNA sequencing

Immunoprecipitation of the RNP complex (protocol adapted from the ChIP protocol described by the Castilo lab(41))

Day 0: Pre-plating cells for transfection

1 million HEK293T cells were plated in 10 cm dishes for transfection the next day.

Day 1: Transfection of the prime editor components

10ug of PEmax expression plasmid and 5ug of pegRNA/epegRNA/sgRNA expression plasmid were transfected using lipofectamine 3000 (Thermofisher scientific) as per manufacturer's instructions.

Day 3: Harvesting the cells and crosslinking

1. 48 hours post transfection, the cells were trypsinized and the pellets were collected at 1000g for 10 minutes at 4°C.
2. Resuspend the cells in 40ml cell culture medium (1% formaldehyde, without FBS) in a 50ml falcon tube for crosslinking.
3. Swirl the falcon tube briefly and let them sit in a shaker with slow rotation at room temperature for 20 minutes.
4. Add 1/20 volume of 2.5 M glycine to the falcon tubes to quench formaldehyde.
5. Spin cells at 1000g for 5 minutes at 4°C.
6. Resuspend the cells in 50ml 1xPBS, spin at 1000g for 5 minutes at 4°C. Discard supernatant. Repeat once.
7. Store cell pellets at –80°C or proceed to the next step.

Lysis and immunoprecipitation

1. Lyse cells using Pierce™ IP Lysis Buffer (Thermofisher scientific #87788).
2. Spin lysate at 20,000g for 10 minutes at 4°C to pellet debris.
3. The supernatant containing the cell lysate was collected and anti-HA tag antibody - ChIP Grade (Abcam #ab9110) was used for immunoprecipitation.
4. Gently mix overnight on a tube rotator at 4°C.

Day 4: Binding to magnetic beads

1. Add 100 µl Dyna magnetic beads (Life technologies, 10004D) to a new microcentrifuge tube. Add 1 ml block solution. Set up 1 tube per IP.
2. Collect the beads using a magnetic stand. Remove the supernatant.
3. Wash beads in 1.0 ml blocking solution two more times.
4. Resuspend beads in 100 µl block solution and add this to the cell lysate+antibody mix from the previous day.
5. Incubate 5-6 hours on a rotating platform at 4°C.

Blocking Solution

Final Conc.	Stock	For 100 ml
1x	10x PBS	10.0 ml
0.5% BSA (w/v)	BSA	500.0 mg
	ddH_2O	90.0 ml
		100.0 ml

Wash, elution, and cross-link reversal

Steps 1 through 6 should be performed in a 4°C cold room.

1. Pre-chill one 1.5 ml microfuge tube for each IP. Let tubes sit in a magnetic stand to collect the beads.
2. Add 1 ml Wash Buffer (RIPA) to each tube. Remove tubes from the magnetic stand and shake or agitate the tube gently to resuspend beads. Replace tubes in the magnetic stand to collect the beads and remove supernatant. Repeat this wash 3 more times.
3. Wash once with 1 ml TE that contains 50 mM NaCl.
4. Spin at 960 x g for 3 minutes at 4°C and remove any residual TE buffer.
5. Add 210 µl of elution buffer.
6. Elute at 65°C for 60 minutes at 1000rpm (in a thermo mixer). Resuspend beads every 10 minutes by brief vortexing.
7. Spin down beads at 16,000 x g for 1 minute at room temperature.
8. Remove 200 µl of supernatant and transfer to new tube. Reverse crosslink by incubating at 65°C overnight.

Wash Buffer (RIPA)

Final Conc.	Stock	For 250 ml
50 mM	1M Hepes-KOH, pH 7.6	12.5 ml
500 mM	5M LiCl	25.0 ml
1 mM	0.5M EDTA	0.5 ml
1%	10% NP-40	25.0 ml
0.7%	10% Na-Deoxycholate	17.5 ml
	ddH_2O	169.5 ml

Elution Buffer

Final Conc.	Stock	For 100 ml
50 mM	1M Tris-HCl, pH 8.0	5.0 ml

10 mM	0.5M EDTA	2.0 ml
1%	10% SDS	10.0 ml
	ddH_2O	83.0 ml

Day 3: Digestion of DNA and the immunoprecipitated protein and RNA purification

1. Add 200 µl of TE to each tube of IP to dilute SDS in the elution buffer.
2. Add 1uL of DNAse (M030S) per sample.
3. Mix and incubate at 37°C for 1 hour.
4. Add 4 µl of 20 mg/ml proteinaseK (0.2 µg/ml final concentration).
5. Mix and incubate at 55°C for 1 hour.
6. The recovered RNA was purified using the Monarch® RNA Cleanup Kit (T2050L) as per the kit's protocol. Elute in 100 µL in 1.5ml centrifuge tubes
7. Lyophilize the eluted RNA and then resuspend the pellet in 8µL of nuclease free water. Proceed to the 3' UMI adapter ligation protocol.

3' UMI adapter ligation (Protocol adapted from the illumina TruSeq small RNA library protocol described by the Zamore lab(42))

1. Set up 30µL ligation for each sample:

RNA	8 µL
25uM AppBA3-UMI	1 µL
10x 3' ligation buffer	3 µL
T4 Rnl2tr K227Q (NEB, M0351S)	3 µL
50% PEG8000 (Promega, V3011)	15 µL

2. Incubate at 16°C for 16 hours.
3. Purify the RNA using Monarch® RNA Cleanup Kit (T2050L) as per the kit's protocol.
4. Lyophilize the eluted RNA and then resuspend the pellet in 12µL of nuclease free water. Proceed to the cDNA library synthesis.

Library construction

cDNA library synthesis:

1. **Add the following reagents and mix by pipetting:**

10x AMV RT buffer	2 µL
dNTP mix (10mM each)	3 µL
AMV reverse transcriptase (NEB, 10 units/µL)	2 µL
10 µM BRTP RT primer	1 µL

Resuspended RNA pellet (from the previous step)	12 µL
Total Reaction	20 µL

2. Place tubes in a thermal cycler and run the following program with a heated lid for cDNA synthesis:

(1) 42°C for 1 hr

(2) 90°C for 5 min

(3) 4°C forever

(4) End

3. 1st round PCR with scaffold specific primer and BRTP primer

1 µL sgRNA scaffold library 5p

1 µL BRTP primer

12.5 µL Phusion Flash High-Fidelity PCR Master Mix (2X)

5 µL cDNA synthesis reaction

5.5 µL Nuclease-Free water

Total: 25 µL

Step	Temp (^oC)	Time	Cycle #
Initial Denaturation	98	30 sec	1
Denature	98	10 sec	
Anneal	58	10 sec	30
Extend	72	5 sec	
Final Extension	72	2 min	1
Hold	4		1

4. 2nd round PCR to add 5' and 3' indexes for multiplex amplicon sequencing

PCR set up to add different Barcodes on both 5' and 3' ends for different samplesReagents	1 reaction
2x Phusion Flash High-Fidelity PCR Master Mix	25 µL
i5 barcode primer (10 µM)	2 µL
i7 barcode primer (PCRIdx) (10 µM)	2 µL

1st round PCR product	5 µL
Nuclease-Free water	16 µL
Total reaction	50 µL

Mix well
Place plate in thermocycler and run the following program:

Step	Temp (°C)	Time	Cycle #
Initial Denaturation	98	2 min	1
Denature	98	10 sec	15
Anneal	65	10 sec	
Extend	72	10 sec	
Final Extension	72	2 min	1
Hold	4		1

5. Clean up using Agencourt Ampure XP SPRI bead (0.9X reaction volume)

5.1 Materials:

- Agencourt AMPure XP (#A63882)
- DynaMag™-96 Side Magnet (#12331D)
- 200 Proof Pure Ethanol (KOPTEC #V1016)
- DNase-RNase Free Water Non-DEPC Treated (Boston Bioproducts #R-100DR)

5.2 Ampure XP SPRI beads Cleanup for **2nd round PCR** :

1. Add 45 µL of Ampure XP beads to each tube containg 50ul 2nd round PCR reaction
2. Mix beads with reaction by pipetting up and down 15 times
3. Let stand in room temperature for 5 minutes
4. Place the tube on the magnet and let stand for 5 minutes or until the solution is clear
5. Remove and discard supernatant
6. Wash two times with 200 µL of prechilled 70% ethanol (made fresh before use). Let the ethanol sit in the beads for 30 seconds then remove ethanol
7. Let the beads air dry for 3-5 minutes (until the pellet is not glossy)
8. Elute with 25 µL of 1x low TE (10 mM Tris, 0.1 mM EDTA)

6. QC with Bioanalyzer/TapeStation and Qubit

6.1 Bioanalyzer QC following manufacturer's specifications
http://www.agilent.com/cs/library/usermanuals/Public/G2938-90322_HighSensitivityDNA_QSG.pdf

6.2 Qubit Quantification following manufacturer's specifications for the dsDNA High Sensitivity kit
https://tools.thermofisher.com/content/sfs/manuals/Qubit_dsDNA_HS_Assay_UG.pdf

7. Pool, double size select, and concentrate samples

7.1 Materials

- Agencourt AMPure XP (#A63882)
- DynaMag™-96 Side Magnet (#12331D)
- 200 Proof Pure Ethanol (KOPTEC #V1016)
- DNase-RNase Free Water Non-DEPC Treated (Boston Bioproducts #R-100DR)

7.2 Pooling and SPRI clean up

1. Calculate the molar amount of each product based on the concentration from Qubit and the size from the TapeStation.
2. Pool equimolar amounts of each product into a 1 mL Eppendorf tube.
3. Size select and concentrate pool using Agencourt Ampure XP SPRI beads
    1. Add 0.5X the reaction volume of Ampure XP beads
    2. Mix beads with reaction by pipetting up and down 15 times
    3. Let stand at room temperature for 5 minutes
    4. Place the tube on the magnet and let stand for 5 minutes or until the solution is clear
    5. Remove the supernatant
    6. Wash two times with 200 µL of 70% ethanol (made fresh before use). Let the ethanol sit in the beads for 30 seconds then remove ethanol
    7. Let the beads air dry for 3-5 minutes (until the pellet is not glossy)
    8. Elute with 35 µL of 1x low TE

8. Quantification of final size-selected library using Qubit and Bioanalyzer

1. Quantify library using Qubit dsDNA HS kit
2. Use median size from PCR 2 TapeStation run and Qubit quantitation to determine library concentration

9. MiniSeq Sequencing

9.1 Follow manufacturer's specifications for loading library on MiniSeq

1. Load 1.4 pM denatured library with 30% PhiX
2. Generate MiniSeq run sheet:
    a. Read 1: 150 bases
    b. Index read 1: 8 bases
    c. Read 2: 150 bases
    d. Index read 2: 8 bases

Primers used for small RNA library construction:

	3′ ligation adapter (AppBA3-UMI, 36-nt custom DNA oligo with 5′ adenylation	5′-rAppNNNGTCNNNTAGNNNTGGAATTCTCGGGTGCCAAGG/ddC/-3′

	and 3′ ddC, HPLC purified)	
	RT primer (BRTP, 21-nt custom DNA oligo):	5′-CCTTGGCACCCGAGAATTCCA-3′
	sgRNA scaffold library 5p	5′-ctacacgacgctcttccgatctGCAAGTTAAAATAAGGCTAGTCCGTTATC-3′
i5 barcode primer	i501	AATGATACGGCGACCACCGAGATCTACACTATAGCCTACACTCTTTCCCTACACGACGCTCTTCCGATCT
	i502	AATGATACGGCGACCACCGAGATCTACACATAGAGGCACACTCTTTCCCTACACGACGCTCTTCCGATCT
	i503	AATGATACGGCGACCACCGAGATCTACACCCTATCCTACACTCTTTCCCTACACGACGCTCTTCCGATCT
	i504	AATGATACGGCGACCACCGAGATCTACACGGCTCTGAACACTCTTTCCCTACACGACGCTCTTCCGATCT
i7 barcode primer	PCR Index primer 1 (PCRId1, 63-nt custom DNA oligo, BARCODE):	5′-CAAGCAGAAGACGGCATACGAGATCGTGATGTGACTGGAGTTCCTTGGCACCCGAGAATTCCA-3′
	PCR Index primer 2 (PCRId2, 63-nt custom DNA oligo, BARCODE):	5′-CAAGCAGAAGACGGCATACGAGATACATCGGTGACTGGAGTTCCTTGGCACCCGAGAATTCCA-3′
	PCR Index primer 3 (PCRId3, 63-nt custom DNA oligo, BARCODE):	5′-CAAGCAGAAGACGGCATACGAGATGCCTAAGTGACTGGAGTTCCTTGGCACCCGAGAATTCCA-3′
	PCR Index primer 4 (PCRId4, 63-nt custom DNA oligo, BARCODE):	5′-CAAGCAGAAGACGGCATACGAGATTGGTCAGTGACTGGAGTTCCTTGGCACCCGAGAATTCCA-3′
	PCR Index primer 5 (PCRId5, 63-nt custom DNA oligo, BARCODE):	5′-CAAGCAGAAGACGGCATACGAGATCACTGTGTGACTGGAGTTCCTTGGCACCCGAGAATTCCA-3′
	PCR Index primer 6 (PCRId6, 63-nt custom DNA oligo, BARCODE):	5′-CAAGCAGAAGACGGCATACGAGATATTGGCGTGACTGGAGTTCCTTGGCACCCGAGAATTCCA-3′

	PCR Index primer 7 (PCRId7, 63-nt custom DNA oligo, BARCODE):	5′-CAAGCAGAAGACGGCATACGAGATGATCTGGTGACTGGAGTTCCTTGGCACCCGAGAATTCCA-3′
	PCR Index primer 8 (PCRId8, 63-nt custom DNA oligo, BARCODE):	5′-CAAGCAGAAGACGGCATACGAGATTCAAGTGTGACTGGAGTTCCTTGGCACCCGAGAATTCCA-3′

Supplementary note

RNP complex formation:

Add the pegRNA to the nucleofection buffer first and then add the PE protein gradually to the diluted pegRNA. Allow them to complex at room temperature for 20 minutes before electroporating them into the target cells.

For the PE2 system, we show that the ideal ratio between the protein to the pegRNA is 1:4 or 1:6. However since chemically synthesized pegRNAs are challenging to produce and expensive, we recommend using PE protein : pegRNA at a ratio of 1:4 to achieve high editing rates while reducing the cost of the reaction.

For the PE3 system, we show that while forming a functional RNP complex, the sgRNA outcompetes the pegRNA for binding sites and therefore it is necessary to use them at a ratio where the sgRNA does not saturate binding to the prime editor to the exclusion of pegRNA. We recommend using protein to nicking sgRNA at a ratio of 10:3 or 5:3, while maintaining the protein to pegRNA ratio at 1:4.
